# Effective Components of School-Based Prevention Programs for Child Abuse: A Meta-Analytic Review

**DOI:** 10.1007/s10567-021-00353-5

**Published:** 2021-06-04

**Authors:** Jeanne Gubbels, Claudia E. van der Put, Geert-Jan J. M. Stams, Mark Assink

**Affiliations:** Research Institute of Child Development and Education, University of Amsterdam, Nieuwe Achtergracht 127, P.O. Box 15780, 1018 WS Amsterdam, Netherlands

**Keywords:** School-based programs, Child abuse, Prevention, Meta-analysis, Program components

## Abstract

School-based programs seem promising for child abuse prevention. However, research mainly focused on sexual child abuse and knowledge is lacking on how individual program components contribute to the effectiveness of school-based prevention programs for any form of child abuse. This study aimed to examine the overall effect of these school-based programs on (a) children’s child abuse-related knowledge and (b) self-protection skills by conducting two three-level meta-analyses. Furthermore, moderator analyses were performed to identify how program components and delivery techniques were associated with effectiveness. A literature search yielded 34 studies (158 effect sizes; *N* = 11,798) examining knowledge of child abuse and 22 studies (99 effect sizes; *N* = 7804) examining self-protection skills. A significant overall effect was found of school-based programs on both knowledge (*d* = 0.572, 95% CI [0.408, 0.737], *p* < 0.001) and self-protection skills (*d* = 0.528, 95% CI [0.262, 0.794], *p* < 0.001). The results of the first meta-analysis on children’s child abuse knowledge suggest that program effects were larger in programs addressing social–emotional skills of children (*d* = 0.909 for programs with this component versus *d* = 0.489 for programs without this component) and self-blame (*d* = 0.776 versus *d* = 0.412), and when puppets (*d* = 1.096 versus *d* = 0.500) and games or quizzes (*d* = 0.966 versus *d* = 0.494) were used. The second meta-analysis on children’s self-protections skills revealed that no individual components or techniques were associated with increased effectiveness. Several other study and program characteristics did moderate the overall effects and are discussed. In general, school-based prevention programs show positive effects on both knowledge and self-protection skills, and the results imply that program effectiveness can be improved by implementing specific components and techniques.

**Supplementary Information:**

The online version contains supplementary material available at 10.1007/s10567-021-00353-5.

The World Health Organization states that “child abuse or maltreatment constitutes of all forms of physical and/or emotional ill-treatment, sexual abuse, neglect or negligent treatment or commercial or other exploitation, resulting in actual or potential harm to the child’s health, survival, development or dignity in the context of a relationship of responsibility, trust or power” (World Health Organization, 1999). Worldwide, child abuse is a major public health problem that can have long lasting negative effects for children, such as physical, behavioral, and psychological problems, and that contributes substantially to child mortality and morbidity (Alink et al., 2012; Cicchetti, 2016; Jonson-Reid et al., 2012). Associations were also found between child abuse victimization and problems in multiple domains of functioning, such as academic achievement, social and emotional development, psychopathology, and neurobiological deficits (Widom, 2014). A series of meta-analyses on the worldwide prevalence of child abuse showed an overall estimated prevalence ranging from 12.7% (for sexual abuse) to 36.3% (for emotional abuse) in self-report studies, and a prevalence ranging from 0.3% (for physical abuse and emotional abuse) to 0.4% (for sexual abuse) in studies using informants (Stoltenborgh et al., 2015). Given the high prevalence rates of child abuse and the serious short-term and long-term negative effects on children’s well-being, effective prevention of child abuse is essential.

The implementation of school-based programs is a promising approach to child abuse prevention. Most children daily attend school which provides opportunities for teachers and other school staff to detect child abuse risk factors (Citak Tunc et al., 2018; Daigneault et al., 2012; Nickerson et al., 2019). Review studies showed that school-based prevention programs increase a child’s knowledge, self-protection skills, and the likelihood of abuse disclosure (Davis & Gidycz, 2000; MacIntyre & Carr, 2000; Rispens et al., 1997; Topping & Barron, 2009; Walsh et al., 2018). There are also indications that the participation in school-based child abuse prevention programs is associated with reduced child abuse rates (Gibson & Leitenberg, 2000). Studies on the effectiveness of school-based programs mainly focused on the prevention of child sexual abuse even though this is the least common form of child abuse (Stoltenborgh et al., 2015). Little is known about the effects of school-based prevention programs for any form of child abuse, including neglect and physical abuse. Knowledge is also lacking on how specific program components and delivery techniques are related to the effectiveness of school-based child abuse prevention programs. This knowledge is important to further improve the effectiveness of these programs. Therefore, the aim of this meta-analytic review was twofold: (1) to examine the effectiveness of school-based programs for the prevention of (any form of) child abuse, and (2) to explore how individual study and program characteristics are associated with program effectiveness, including program components and delivery techniques.

## School-Based Child Abuse Prevention Programs

In general, school-based prevention programs aim to prevent child abuse by providing children child abuse-related knowledge and self-protection skills that decrease a child’s risk for abuse (Blakey & Thigpen, 2015). In this review, child abuse-related knowledge refers to knowledge on child abuse and prevention-related concepts, such as the different types of abuse, unsafe secrets, and inappropriate touch. This knowledge might enable children to recognize abuse or unsafety, both in their own situation and in the situation of their peers. Self-protection skills refer to protective strategies that a child can use to protect itself from abuse or strategies that reduce the overall level of child abuse risk, such as saying ‘no,’ finding help and avoiding or escaping unsafe situations. The assumption is that increased knowledge and skills make children more aware of abusive or unsafe situations, and make them more assertive and less compliant with offenders (Ko & Cosden, 2001). In addition, children attending school-based prevention programs are encouraged to disclose potential abuse to a friend or an adult they trust. This may prevent unsafe situations from deteriorating or stop ongoing abuse and it may help child welfare services to get in contact with children and families sooner (Baker et al., 2012). Often covered topics by these programs include recognizing and avoiding abusive situations, seeking help, identifying and disclosing abuse to trusted adults, understanding body ownership, and having the skills to say ‘no’ and/or escape abusive situations (MacIntyre & Carr, 2000; Nickerson et al., 2019). The school-based prevention programs differ in the number and type of topics covered, and vary on a number of other dimensions, such as the way in which a program is delivered, the age of the participating children, type of program leader, and the length of the program (Davis & Gidycz, 2000).

School-based child abuse prevention programs also differ in the type of abuse that they target. Since the 1980s, school-based programs have been adopted as a popular method for the prevention of child sexual abuse (Berrick & Gilbert, 1991; Daro, 1991). An example of such a program is the Behavioral Skills Training Program (BST; Wurtele, 1986). In this program, children learn personal safety skills from a behavioral perspective. In small groups, teachers address several safety aspects, including that children are the owners (“bosses”) of their bodies and that it is not right to have their private parts touched or looked at by an adult person. This program was evaluated in two randomized controlled trials in which low-income preschool children participated (Wurtele, Gillispie, et al., 1992; Wurtele, Kast, et al., 1992). In both trials, children in the BST group demonstrated greater knowledge about sexual abuse and higher levels of personal safety skills compared to those in the control group. These gains were maintained at both 2-month (Wurtele, Gillispie, et al., 1992) and 5-month (Wurtele et al., 1992a; Wurtele, Kast, et al., 1992) follow-ups.

Although many school-based child abuse prevention programs focus exclusively on sexual abuse, various programs also address other forms of child abuse, such as physical abuse, emotional abuse, or neglect. An example of a school-based prevention program for physical and sexual abuse is the Play is Safe! program. This program was created by the Women’s Center of Tarrant County in 1983 and teaches children to recognize abusive situations, how to respond to potentially abusive situations, and to report the abuse to someone who can help stop the abuse. Blakey and Thigpen (2015) evaluated the Play it Safe! program in a pre-posttest study and found overall gains in physical and sexual abuse knowledge scores.

The school-based approach to child abuse prevention programs has several advantages. First, schools are ideal settings for program delivery as they provide access to the general population and many children can be reached in a relatively cost-efficient manner (Asawa et al., 2008; Dhooper & Schneider, 1995). Research also showed that children consider education at school to be an important strategy for preventing child abuse and neglect (Pieper & De Haan, 2017). School-based programs are often delivered by teachers or other school staff who can enhance learning experiences regarding child abuse prevention and recognize potential child abuse because of their daily interactions with the children (Nickerson et al., 2010, 2019). Children may also feel more comfortable reporting suspected child abuse to their teacher if their teacher delivers the school-based program and opens the dialog. Teachers and school staff are often considered “trusted adults” to children and delivery by a teacher may be particularly important when a prevention program includes parent participation when a parent is abusive. However, delivery by teachers has been criticized as, despite their pedagogical competence, they may not have sufficient knowledge and experience, nor the confidence to talk with children about the specific topics covered by a child abuse prevention program (Topping & Barron, 2009). Finkelhor (2007) has also addressed several concerns about school-based child abuse prevention programs that arose over the years, for instance that the concepts covered by these programs are too complex for children to grasp. He also noted that abuse cannot be prevented or deterred by the actions of children themselves, as many children are too weak and too vulnerable to resist perpetrators who are often older, larger, and aggressive. Finkelhor (2007) did, however, conclude that it is worth providing children with high-quality prevention programs, as this is supported by current scientific evidence.

## Previous Review Studies

Several meta-analyses and systematic reviews on the effects of school-based prevention programs specifically for child sexual abuse found significant improvements in abuse-related knowledge and self-protection skills. For example, Rispens et al. (1997) found an overall post-intervention effect of *d* = 0.71 and a follow-up effect of *d* = 0.62 of school-based victimization prevention programs on children’s self-protection skills and knowledge of sexual abuse concepts. Davis and Gidycz (2000) found a slightly larger effect of *d* = 1.07 for these outcomes. More recently, Walsh et al. (2018) found a Cohen’s *d* of 0.61 and 0.45 for factual and applied knowledge of sexual abuse and prevention concepts, respectively, and *d* = 0.96 for protective behaviors of children. They also examined the degree to which sexual abuse was disclosed by children and whether the program caused harm, manifested as parental or child anxiety or fear, but no effects were found for these outcomes. Furthermore, a significant effect of school-based child sexual abuse prevention programs on the occurrence of child sexual abuse was found in a retrospective study (Gibson & Leitenberg, 2000). Young women who had not participated in a school prevention program during their childhood were about twice as likely (*OR* = 2.11) to have experienced child sexual abuse as those who had participated in such a program. Overall, the literature suggests that school-based programs may contribute to the prevention of child abuse.

Davis and Gidycz (2000) as well as Rispens et al. (1997) explored potential moderating variables of the effect of school-based programs. In both reviews, stronger effects were found for relatively younger children, programs of longer duration or more sessions, and programs based on hands-on training of behavioral skills. Additionally, Davis and Gidycz (2000) found stronger effects when programs actively involved children in the sessions and when the outcome was measured by means of behavioral observations. However, it is important to examine how specific content components of school-based prevention programs are associated with overall program effectiveness for gaining more knowledge on what influences the effectiveness of school-based prevention programs and to determine why some programs are more effective than other. Moreover, identifying effective components is essential for developing or improving school-based programs. In reviewing existing sexual abuse education programs, Kenny et al. (2008) found some key components of successful programs. Examples of these essential components are teaching children how to identify and resist inappropriate touching, reassuring children that abuse is not their fault, and learning the proper names of their genitals. Although this type of research sheds some light on key components of school-based prevention programs, quantitative research on the potential moderating effect of these components is still lacking.

## The Current Study

To the best of our knowledge, this is the first meta-analytic review examining school-based programs for the prevention of any form of child abuse. Previous meta-analyses solely focused on the effectiveness of school-based programs for sexual abuse. However, research showed that sexual abuse is the least prevalent form of worldwide self-reported child maltreatment (12.7%) compared to, for example, physical abuse (22.6%) and emotional abuse (36.3%; Stoltenborgh et al., 2015). Therefore, the effectiveness of school-based programs aimed at preventing any form of child abuse, rather than solely sexual abuse, should be examined. Furthermore, this is the first study to examine the possible moderating effect of specific components and techniques of school-based programs. This will enhance knowledge on how these components are associated with effectiveness of school-based prevention programs, which provides insight into how these programs can be improved. Finally, this meta-analytic review improves prior reviews by using a three-level meta-analytic technique. With this three-level approach it is possible to include all relevant effects reported in each primary study, implying that all relevant information is preserved. As a result, no information is lost and (moderator) effects can be estimated more precisely and with maximum statistical power (Assink & Wibbelink, 2016).

To summarize, this study aimed to meta-analytically summarize empirical evidence for the effectiveness of school-based child abuse prevention programs. Therefore, we conducted two three-level meta-analyses to examine the overall effect of school-based programs on two outcomes, namely (1) child abuse-related knowledge and (2) children’s self-protection skills (including abuse disclosure). Furthermore, we examined the contribution of study and program characteristics to this effectiveness, including program components and delivery techniques, by conducting moderator analyses.

## Method

### Inclusion Criteria

To be included in the current meta-analysis, primary studies had to meet the following inclusion criteria. First, studies had to report on the effect of at least one school-based child abuse prevention program on child abuse-related knowledge and/or self-protection skills of children. School-based prevention programs were defined as programs implemented by an instructor (e.g., teacher, school nurse, external instructor) in a school setting, which are aimed at preventing child abuse by providing children knowledge and self-protection skills that decreases a child’s risk for abuse.

For the concept of child abuse, we followed the definition as formulated by the World Health Organization (also see the Introduction): “child abuse or maltreatment constitutes of all forms of physical and/or emotional ill-treatment, sexual abuse, neglect or negligent treatment or commercial or other exploitation, resulting in actual or potential harm to the child’s health, survival, development or dignity in the context of a relationship of responsibility, trust or power” (World Health Organization, 1999). Following this definition, we included studies that reported on any form of child abuse (i.e., physical abuse, sexual abuse, emotional abuse, and any form of neglect) committed by parents or caregivers as well as other adults or people with whom there is a relationship of responsibility, trust, or power. Studies reporting on extrafamilial child (sexual) abuse were therefore also included in this meta-analysis. As for child abuse-related knowledge, studies had to measure knowledge on child abuse and prevention-related concepts (e.g., knowing about or recognizing different types of abuse, safety rules, unsafe secrets, inappropriate touch), as measured by questionnaires (e.g., Children’s Knowledge of Abuse Questionnaire; CKAQ; Tutty, 1995) or vignettes (tools based on the ‘What If’ Situation Test; WIST; Wurtele et al., 1998). As for self-protection skills, studies had to measure protective behaviors that protect a child from abuse (e.g., saying ‘no,’ getting help, telling a friend, escaping unsafe situations). Disclosing child abuse during or after the program or outcomes related to disclosure (i.e., disclosure intentions or confidence to disclose) were also considered protective behaviors. Self-protection skills were measured with questionnaires (i.e., mostly tools designed authors of primary studies), vignettes (i.e., the WIST), or in-vivo simulations (i.e., Observed Protective Behaviors Test; OPBT; White et al., 2018).

Second, only studies in which a treatment condition was compared to a control condition were included, implying that both experimental and quasi-experimental studies were included. Finally, primary studies had to report on at least one effect size or sufficient information to calculate an effect size.

### Study Selection

**Fig. 1:**
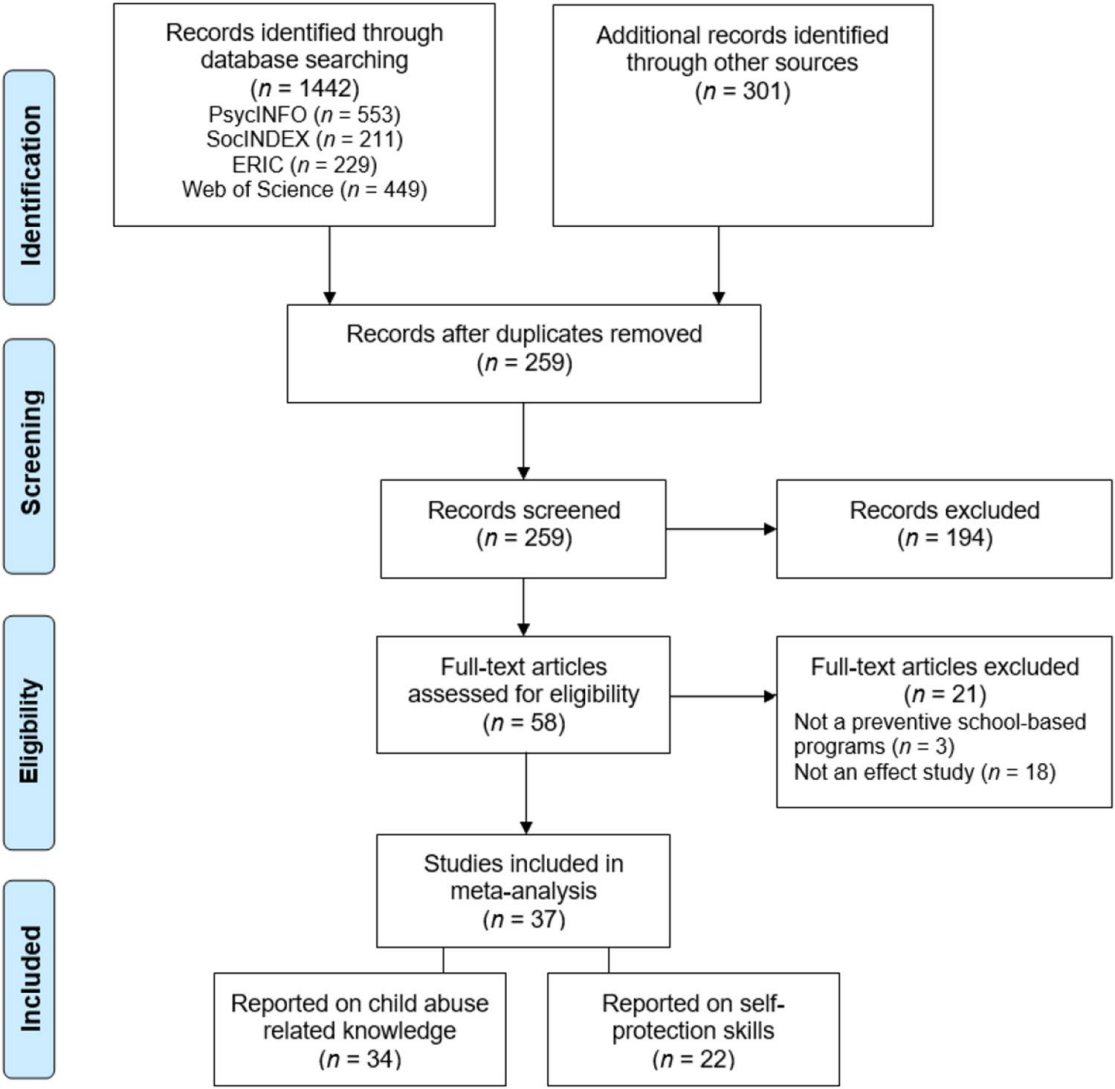
Flowchart of study selection procedure, according to the Preferred Reporting Items for Systematic Review and Meta-Analysis (PRISMA)

A comprehensive search strategy was carried out to identify and retrieve all relevant studies. First, several electronic databases were searched for relevant articles, reports, dissertations, books, and book chapters. For the search syntax and all keywords used in this electronic search, see Appendix A (Supplementary Material). Second, the full reference lists of all included primary studies as well as various relevant meta-analyses and systematic reviews were searched (i.e., Davis & Gidycz, 2000; MacIntyre & Carr, 2000; Rispens et al., 1997; Topping & Barron, 2009; Walsh et al., 2018). This search strategy resulted in 1743 studies. After removing duplicates, 249 studies were screened based on their title and/or abstract. In the screening phase, 194 studies were excluded because of their irrelevance to the subject of this meta-analysis. Of the remaining 58 studies, the full text was evaluated. Finally, 37 studies met all inclusion criteria and were included in the current study, with 34 studies reporting on child abuse-related knowledge and 22 reporting on self-protection skills. A flow chart of the search procedure is presented in Fig. 1. The characteristics of included studies are presented in Appendix B (Supplementary Material).

### Coding the Studies

A coding scheme was designed using the guidelines proposed by Lipsey and Wilson (2001) to code relevant study and program characteristics that could moderate the effect of school-based child abuse programs. As for the study characteristics, we coded publication year and the continent in which the study was performed (North-America, Europe, Australia, Asia, Other). The other characteristics were classified into sample, design, and outcome characteristics. The coded sample characteristics were the sample size, mean age of the child (in years), type of school (on which the program was delivered; elementary school, preschool/kindergarten, high school), mean age of the parents (in years), percentage of girls in the sample, and percentage of non-Caucasians/non-Whites in the sample. The coded design characteristics were type of experiment (RCT, cluster RCT, quasi-experimental), intent-to-treat analysis (yes/no), whether or not the program fidelity was monitored or measured (not reported/monitored, only monitored, monitored, and measured), type of program in control group (no program, waiting list), and whether or not group differences at baseline were measured (yes/no). Finally, type of outcome (knowledge, skills, disclosure), type of outcome measurement (questionnaire, vignettes, in-vivo simulation), and the follow-up period (in months) regarding an outcome were coded as outcome characteristics. Based on the outcomes that were examined in the primary studies, each extracted effect size was included in (a) the meta-analysis on child abuse knowledge or (b) the meta-analysis on self-protection skills. In the latter, the effect of school prevention programs was examined separately for self-protection skills and disclosure outcomes.

As for program characteristics, we coded the type of abuse the program was targeting (only sexual child abuse, any form of child abuse), the type of instructor (external, teacher, school nurse/social worker, combination), whether school personnel received training on the program’s concepts (yes/no), whether parents are involved in the program (yes/no), the program duration (in weeks), the number of sessions of the program, the duration of each session (in minutes), and the intensity of the sessions (weekly, more than once a week, only one session, other). We coded 12 specific components on which the content of a program could be based, which were (1) promoting knowledge on child abuse or prevention concepts or definitions, (2) identifying a trusted person (e.g., identify people in family, building a community support system), (3) learning about safe and unsafe secrets (i.e., secrets that are okay or not okay to keep), (4) increasing awareness of children’s personal rights (e.g., rights to be safe, rights over their own body), (5) increasing social–emotional skills (e.g., empathy with peers, social problem-solving skills), (6) teaching children to avoid self-blame (that abuse is never the child’s fault), (7) learning about own body and boundaries (e.g., learning about private parts, safe or unsafe touch), (8) recognizing and avoiding risky situations (e.g., recognize potentially abusive situations or potential abusers), (9) increasing assertiveness skills (e.g., saying ‘no,’ standing up for oneself), (10) learning to go away from a potential abusive situation or to find help, (11) learning skills to disclose abuse (e.g., encouraging children to report abuse to trusted adult, developing the vocabulary needed to report), and (12) increasing a child’s self-esteem.

Finally, we coded the following delivery techniques either as present or absent: (1) group discussion or debate, (2) behavioral skills training techniques (i.e., role-playing, rehearsal, feedback, and/or praise), (3) photos, pictures, or posters (e.g., about abusive situations or private body parts to discriminate appropriate from inappropriate touches), (4) video (e.g., depicting abuse situations, child abuse concepts, or prevention concepts), (5) puppets (which for instance are used for explaining body safety rules), (6) vignettes or stories about potential abusive or dangerous situations, (7) workbook (for instance with assignments about body safety that are to be completed at home with caregivers, or an activity book that can be used in program sessions), (8) modeling (e.g., modeling appropriate behaviors in abusive situations), (9) games or quizzes (e.g., true or false quizzes, online games), and (10) theater play (in which potential abusive situations are presented).

To determine the interrater reliability, 10 studies eligible for inclusion (reporting on a total of 28 effect sizes) were randomly selected and double coded by the first and last author of this study. Inter‐rater agreement was analyzed by calculating the percentage of agreement for all variables, Cohen’s Kappa for categorical variables, and intraclass correlation for continuous variables. As for the study and program characteristics, inter‐rater reliability for categorical variables ranged from *κ* = 0.33 (70% agreement) for intent-to-treat analysis to *κ* = 1.00 (100% agreement) for 7 variables (e.g., type of school). The intraclass correlation coefficients for the continuous study and program variables ranged from 0.68 (80% agreement) for percentage of non-Caucasians/non-Whites to 1.00 (100% agreement) for 8 variables (e.g., age of the child). For the double-coded program components and techniques inter‐rater reliability ranged from *κ* = 0.44 (70% agreement) for recognizing and avoiding risky situations to *κ* = 1.00 (100% agreement) for 9 components and 7 techniques (e.g., teaching children to avoid self-blame). The intraclass correlation coefficient for the double-coded effect sizes was 1.00 (89% agreement). As these statistics indicate there were inconsistencies in coding, all inconsistencies were discussed and resolved until the authors fully agreed on all final coding decisions. When final consensus was reached, all other studies were coded by the first author. Whenever the first author doubted about the coding of a variable for any of the included studies, the other authors were consulted.

### Calculation of Effect Sizes

Each reported relevant effect in one of the included studies was transformed into a Cohen’s *d*, the standardized difference between two means. The statistics reported in the primary studies, including means and standard deviations, proportions, *t* values, and *F* values, were transformed into Cohen’s *d* values using formulas of Ferguson (1966), Lipsey and Wilson (2001), and Rosenthal (1994). As for the direction of effect sizes, a positive *d* value indicated higher levels of child abuse-related knowledge or more self-protection skills in the group that received the preventive school-based program compared to the control group, whereas a negative *d* value indicated that less knowledge or skills was found in the school-based program group than in the control group. Some primary studies reported on the children’s knowledge and/or skills prior to the start of the study. To control for differences in knowledge and/or skills between children in the intervention group and children in the control group, the Cohen’s *d* corresponding to the posttest or follow-up outcomes were reduced by the pre-test Cohen’s *d*. All coded variables and calculated effect sizes were entered in SPSS version 25. Next, continuous variables were centered on their mean, and categorical variables were recoded into dummy variables.

### Statistical Analyses

Two three-level meta-analyses were performed to examine the overall effect of school-based child abuse prevention programs on either child abuse-related knowledge and self-protection skills, and to examine variables with a potential moderating effect. A major advantage of this three-level approach to meta-analysis over a traditional random effect (two-level) model is that there is no need for selecting or aggregating outcomes reported in primary studies, as dependency between outcomes is modeled. This implies that all relevant effect sizes can be extracted from each primary study and maximum statistical power can be achieved (see, for instance, Assink & Wibbelink, 2016). In a three-level random effects meta-analytic model, three sources of variance are taken into account: sampling variance of the observed effect sizes (Level 1), variance between effect sizes extracted from the same study (Level 2), and variance between studies (Level 3; Van den Noortgate et al., 2013; 2015). For estimating the overall effect, we built an intercept-only model without covariates. In this model, the intercept represented the overall effect. If variation in effect sizes extracted from the same study (i.e., level 2 variance) and/or variation in effect sizes extracted from different studies (i.e., level 3 variance) was significant, the model was extended with the potential moderating variables to determine whether these variables can explain any significant variance. We examined the significance of the level 2 and level 3 variance by performing two separate one-tailed log-likelihood-ratio tests.

The program R (version 3.6.1) and the metafor-package (Viechtbauer, 2010) were used to perform all analyses. We used the R syntax as described by Assink and Wibbelink (2016). In all analyses, a 5% significant level was used.

### Publication Bias

A common problem in conducting a meta-analysis is publication bias, also referred to as the ‘file drawer problem’ by Rosenthal (1995), which implies that studies producing non-significant or negative results are less likely to be published than studies producing positive and significant results. Therefore, the studies included in a review may not be an adequate representation of all available studies relevant to a particular subject, and thus the results may be biased. To examine whether the results of the present meta-analyses were affected by (different forms of) bias, we conducted non-parametric and funnel-plot-based trim-and-fill analyses as described by Duval and Tweedie (2000a, 2000b). In a trim-and-fill analysis the symmetry of a funnel-plot is tested, which is a scatter plot in which effect sizes are plotted against their standard error. Bias may be present if the funnel is asymmetric. In case of an asymmetric funnel, the symmetry can be restored by imputing “missing” effect sizes that are estimated on the basis of existing effect sizes in the dataset. Effect sizes imputed to the left of the estimated mean effect imply that below-average effect sizes are underrepresented and that the mean effect may be an overestimation of the true effect. On the other hand, imputation of effect sizes to the right of the estimated mean effect indicates that above-average effect sizes are underrepresented and that the estimated mean effect may be an underestimation of the true effect. After imputing the “missing” effect sizes, an adjusted overall effect can be estimated. In this way, the degree to which the results were affected by bias can be determined. The trim-and-fill analyses were conducted using the “trimfill” function of the “metafor” package (Viechtbauer, 2010) in the program R (version 3.6.1).

## Results

In total, *k* = 37 studies published between 1985 and 2019 were included, with *k* = 34 studies reporting on the effect of school-based programs on child abuse-related knowledge and *k* = 22 studies reporting on the effect of these programs on self-protection skills (including disclosure). For the former, the 34 studies reported on 158 effect sizes and a total of *N* = 11,798 children, of whom *n* = 6608 participated in a school-based prevention program and *n* = 5190 were allocated to a control group. The sample sizes of the included studies varied between *n* = 19 and *n* = 2172. Study participants’ mean age at baseline was 8.8 years (*SD* = 2.45), ranging from 4.1 year to 18.5 years. The included studies were conducted in the USA (*k* = 16), Europe (*k* = 5), Asia (*k* = 6), Canada (*k* = 4), Australia (*k* = 1), Ecuador (*k* = 1), and Nigeria (*k* = 1). As for the latter, the 22 studies reported on 99 effect sizes and a total of *N* = 7804 participants, with study sample sizes varying between *n* = 13 and *n* = 2172. These studies examined *n* = 4290 children participating in a school-based program and *n* = 3514 children were allocated to a control group. The average age of the participating children was 8.0 years (*SD* = 2.48), ranging from 4.1 to 15.1 years. The studies were conducted in the USA (*k* = 9), Europe (*k* = 5), Asia (*k* = 5), Canada (*k* = 2), and Australia (*k* = 1).

### Overall Effect on Knowledge

We found a significant overall effect of school-based child abuse prevention programs on child abuse-related knowledge with a positive effect size of *d* = 0.572; 95% CI [0.408, 0.737], *t* (157) = 6.857, *p* < 0.001 (see Table 1). According to the criteria formulated by Cohen (1988) for the magnitude of effect sizes, with effect sizes of *d* = 0.20 considered small, *d* = 0.50 medium, and *d* = 0.80 large, this effect is medium in magnitude. The two log-likelihood ratio tests showed that significant variance was present both at level 2 (*χ*^2^(1) = 777.4608, *p* < 0.001; one-sided) and level 3 (*χ*^2^(1) = 125.3712, *p* < 0.001; one-sided) of the meta-analytic model. Of the total variance, 4.3%, 52.7%, and 43.0% were distributed at levels 1, 2, and 3, respectively.

**Table 1:** Overall effects for knowledge and skills

	# Studies	# ES	Mean *d* (SE)	95% CI	Sig. mean *d* (*p*)	% Var. at level 1	Level 2 variance	% Var. at level 2	Level 3 variance	% Var. at level 3
Overall effect knowledge	34	158	0.572 (0.083)***	(0.408, 0.737)	< .001***	4.3	0.178***	52.7	0.145***	43.0
Overall effect skills	22	99	0.528 (0.134)***	(0.262, 0.794)	< .001***	4.5	0.121***	26.1	0.322***	69.4

#Studies = number of studies; # ES = number of effect sizes; Mean *d* = mean effect size (Cohen’s *d*); SE = standard error; CI = confidence interval; Sig. = significance; % Var. = percentage of distributed variance; level 1 variance = sampling variance; level 2 variance = variance within studies; level 3 variance = variance between studies

*p < .05; **p < .01; ***p < .001

^+^*p* < .10

**Fig. 2:**
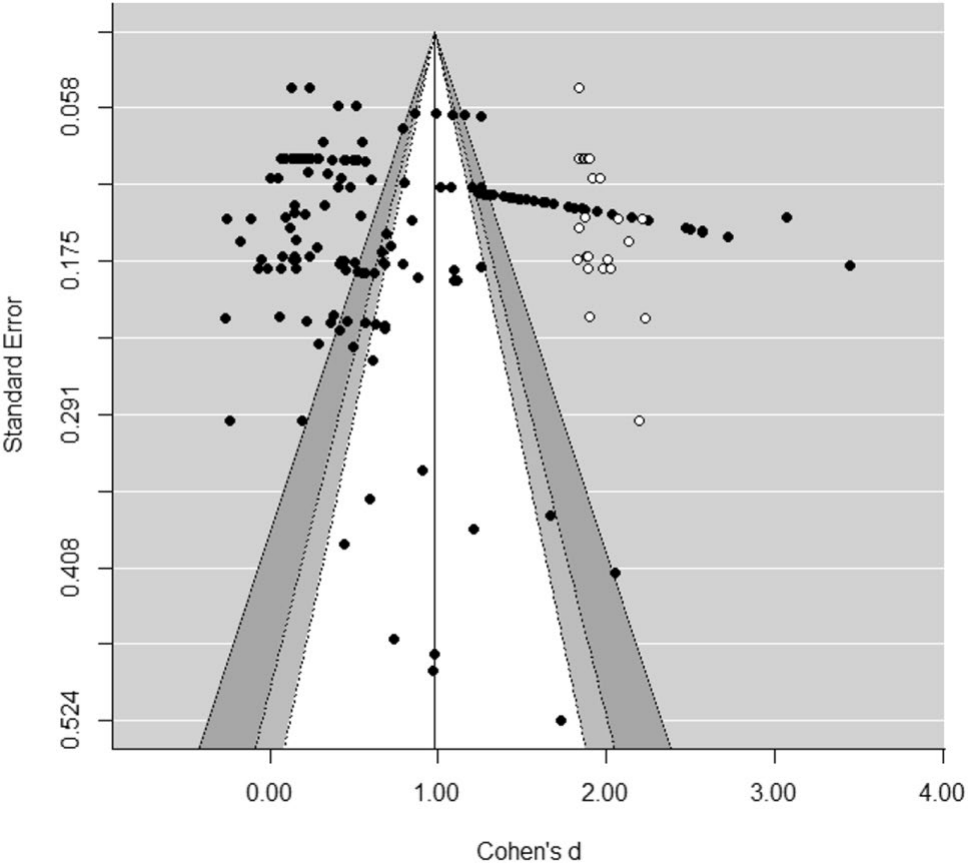
Funnel plot of the trim-and-fill analysis knowledge

The results of the trim-and-fill analysis showed that the distribution of effect sizes was asymmetrical. Figure 2 reveals that in particular large and positive effect sizes were missing in the dataset. As this does not indicate publication bias, we did not estimate a “corrected” overall effect that would be larger than the estimated mean effect of *d* = 0.572.

### Overall Effect on Self-Protection Skills

Table 1 also presents the estimated overall effect of school-based prevention programs on self-protection skills of children. A significant overall effect was found with a Cohen’s *d* of 0.528; 95% CI [0.262, 0.794], *t* (157) = 3.936, *p* < 0.001, which is considered medium in magnitude (Cohen, 1988). Significant variance was present at level 2 (*χ*^2^(1) = 135.6082, *p* > 0.001; one-sided) as well as at level 3 (*χ*^2^(1) = 57.3315, *p* < 0.001; one-sided). Of the total variance, 4.5%, 26.1%, and 69.4% were distributed at levels 1, 2, and 3, respectively.

**Fig. 3:**
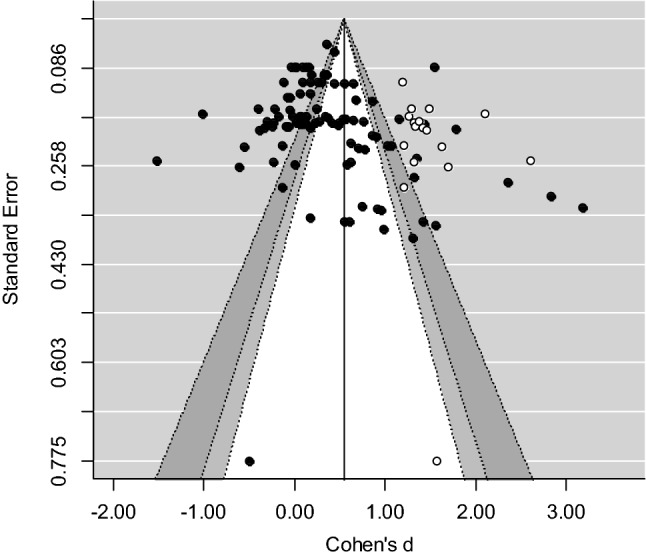
Funnel plot of the trim-and-fill analysis self-protection skills

Figure 3 again shows that in particular large and positive effect sizes were underrepresented. Once again, we did not estimate a “corrected” overall effect.

### Moderator Analyses for Knowledge

Table 2 shows the results of the moderator analyses for child abuse-related knowledge. The potential moderators were classified into study and program characteristics. The latter was further classified into program components and delivery techniques.

**Table 2:** Results of the moderator analyses for knowledge

Moderator variables	# Studies	# ES	Intercept/mean *d* (95% BI)	β_1_(95% CI)	*F* (df1, df2)^a^	*p*^b^	Level 2 variance	Level 3 variance
Overall Effect	34	158	0.572 (0.408, 0.737)***			< 0.001***	0.178***	0.145***
Study characteristics								
Publication year	34	158	0.578 (0.411, 0.745)***	0.004 (−0.010, 0.018)	0.326 (1, 156)	0.569	0.178***	0.147***
Continent					1.714 (3, 153)	0.150	0.179***	0.126***
North America (RC)	21	67	0.490 (0.286, 0.693)***					
Europe	4	52	1.093 (0.665, 1.520)***	0.603 (0.130, 1.076)*				
Australia	1	4	0.360 (−0.461, 1.181)	−0.130 (−0.976, 0.716)				
Asia	6	30	0.550 (0.170, 0.931)**	0.060 (−0.371, 0.492)				
Other^c^	2	5	0.440 (−0.225, 1.106)	−0.050 (−0.745, 0.646)				
Sample characteristics								
Sample size	34	158	0.573 (0.405, 0.740)***	−0.000 (−0.000, 0.000)	0.005 (1, 156)	0.946	0.178***	0.150***
Mean age of child (start study)	30	146	0.564 (0.384, 0.744)***	−0.011 (−0.069, 0.048)	0.128 (1, 144)	0.721	0.191***	0.147***
Type of school					0.441 (2, 155)	0.644	0.179***	0.150***
Elementary school (RC)	29	136	0.588 (0.398, 0.777)***					
Preschool/kindergarten	5	18	0.635 (0.220, 1.050)**	0.047 (−0.403, 0.498)				
High school	1	4	0.314 (−0.268, 0.895)	−0.274 (−0.885, 0.338)				
Mean age of parents (start study)	6	38	0.444 (0.306, 0.582)***	−0.017 (−0.080, 0.045)	0.320 (1, 36)	0.575	0.072***	0.008
Percentage girls	32	153	0.606 (0.435, 0.777)***	−0.178 (−1.508, 1.152)	0.070 (1, 151)	0.792	0.183***	0.144***
Percentage non-Caucasians/non-whites	22	117	0.663 (0.435, 0.891)***	−0.436 (−1.058, 0.186)+	1.926 (1, 116)	0.168	0.209***	0.151***
Design characteristics								
Type of experiment					0.474 (2, 155)	0.624	0.178***	0.153***
Quasi-experimental (RC)	17	114	0.638 (0.406, 0.871)***					
RCT	7	19	0.580 (0.200, 0.960)**	−0.058 (−0.504, 0.387)				
Cluster RCT	10	25	0.446 (0.132, 0.760)*	−0.192 (−0.583, 0.198)				
Intent-to-treat analysis					1.721 (1, 156)	0.192	0.178***	0.141***
No/unknown (RC)	18	112	0.671 (0.451, 0.892)***					
Yes	16	46	0.453 (0.210, 0.696)**	−0.218 (−0.546, 0.110)				
Program fidelity					0.351 (1, 155)	0.704	0.178***	0.153***
Not reported/monitored (RC)	22	128	0.619 (0.412, 0.827)***					
Only monitored	3	9	0.566 (−0.006, 1.138)+	−0.053 (−0.662, 0.555)				
Monitored and measured	9	21	0.454 (0.124, 0.784)**	−0.165 (−0.555, 0.224)				
Type of program in control group					1.047 (1, 132)	0.308	0.142***	0.160***
No program (RC)	12	81	0.698 (0.417, 0.978)***					
Waiting list	22	53	0.513 (0.290, 0.735)***	−0.185 (−0.543, 0.173)				
Group differences at baseline					0.228 (1, 156)	0.633	0.178***	0.152***
No (RC)	12	65	0.624 (0.352, 0.896)***					
Yes	22	93	0.549 (0.327, 0.753)***	−0.084 (−0.429, 0.262)				
Outcome characteristics								
Assessment type					3.530 (1, 156)	0.062+	0.174***	0.147***
Questionnaire (RC)	27	139	0.613 (0.442, 0.783)***					
Vignettes	7	19	0.332 (0.031, 0.633)*	−0.280 (−0.575, 0.014)+				
Follow-up period (in months)	10	44	0.661 (0.198, 1.123)**	−0.051 (−0.300, 0.198)	0.172 (1, 42)	0.680	0.143***	0.343***
Program characteristics								
Type of abuse					0.725 (1, 156)	0.396	0.179***	0.141***
Only sexual child abuse (RC)	26	90	0.534 (0.348, 0.721)***					
Any form of child abuse	8	68	0.702 (0.361, 1.042)***	0.167 (−0.221, 0.556)				
Type of instructor					1.983 (3, 154)	0.119	0.183***	0.107***
External (RC)	16	56	0.459 (0.234, 0.683)***					
Teacher	10	46	0.587 (0.317, 0.857)***	0.129 (−0.222, 0.480)				
School nurse/social worker	5	13	0.559 (0.155, 0.963)**	0.101 (−0.361, 0.563)				
Combination	3	43	1.107 (0.631, 1.583)***	0.649 (0.123, 1.174)*				
Training school personnel					0.424 (1, 56)	0.517	0.058***	0.069***
No/unknown (RC)	9	41	0.488 (0.214, 0.761)***					
Yes	7	17	0.600 (0.390, 0.810)***	0.112 (−0.233, 0.457)				
Are parents involved?					3.122 (1, 156)	0.079+	0.178***	0.123***
No (RC)	25	92	0.482 (0.295, 0.670)***					
Yes	9	66	0.787 (0.503, 1.072)***	0.305 (−0.036, 0.646)+				
Program duration (in weeks)	32	148	0.858 (0.703, 1.014)***	0.054 (0.033, 0.075)***	26.729 (1, 146)	< 0.001***	0.192***	0.047**
Number of sessions	33	156	0.705 (0.523, 0.887)***	0.059 (0.008, 0.109)*	5.316 (1, 154)	0.022*	0.182***	0.110***
Duration of sessions (in minutes)	30	143	0.568 (0.381, 0.754)***	−0.005 (−0.015, 0.004)	1.137 (1, 141)	0.288	0.190***	0.163***
Intensity sessions					2.172 (2, 145)	0.118	0.186***	0.135***
Weekly (RC)	12	99	0.759 (0.499, 1.019)***					
More than once a week	11	36	0.589 (0.310, 0.869)***	−0.169 (−0.551, 0.213)				
Only one session	9	13	0.293 (−0.064, 0.650)	−0.466 (−0.907, −0.024)*				
Program components								
(1) Knowledge concepts/definitions					0.351 (1, 156)	0.555	0.178***	0.147***
No (RC)	16	76	0.523 (0.289, 0.757)***					
Yes	18	82	0.623 (0.387, 0.858)***	0.099 (−0.232, 0.431)				
(2) Identifying trust person					1.132 (1, 156)	0.289	0.179***	0.139***
No (RC)	21	58	0.500 (0.289, 0.712)***					
Yes	13	100	0.679 (0.424, 0.933)***	0.178 (−0.153, 0.509)				
(3) Learning about secrets					0.550 (1, 156)	0.459	0.177***	0.152***
No (RC)	19	95	0.516 (0.291, 0.740)***					
Yes	15	63	0.642 (0.391, 0.894)***	0.127 (−0.211, 0.464)				
(4) Increasing awareness of personal rights					0.494 (1, 156)	0.483	0.179***	0.145***
No (RC)	26	89	0.541 (0.353, 0.728)***					
Yes	8	69	0.681 (0.334, 1.029)***	0.140 (−0.254, 0.535)				
(5) Increasing social–emotional skills					4.852 (1, 156)	0.029*	0.179***	0.113***
No (RC)	28	102	0.489 (0.318, 0.660)***					
Yes	6	56	0.909 (0.574, 1.244)***	0.420 (0.043, 0.796)*				
(6) Teaching to avoid self-blame					5.290 (1, 156)	0.023*	0.177***	0.121***
No (RC)	18	81	0.412 (0.204, 0.621)***					
Yes	16	77	0.776 (0.544, 1.008)***	0.364 (0.051, 0.676)*				
(7) Learning about own body and boundaries					0.951 (1, 156)	0.331	0.178***	0.145***
No (RC)	5	9	0.356 (−0.114, 0.825)					
Yes	29	149	0.603 (0.427, 0.779)***	0.248 (−0.254, 0.749)				
(8) Recognizing and avoid risky situations					0.299 (1, 156)	0.663	0.178***	0.147***
No (RC)	20	60	0.536 (0.312, 0.760)***					
Yes	14	98	0.617(0.370, 0.864)***	0.081 (−0.252, 0.414)				
(9) Increasing assertiveness skills					1.184 (1, 156)	0.278	0.179***	0.140***
No (RC)	10	26	0.428 (0.118, 0.738)**					
Yes	24	132	0.629 (0.437, 0.821)***	0.201 (−0.164, 0.565)				
(10) Learning to go away or to find help					0.748 (1, 156)	0.389	0.179***	0.143***
No (RC)	18	41	0.497 (0.260, 0.735)***					
Yes	16	117	0.641 (0.414, 0.868)***	0.144 (−0.185, 0.473)				
(11) Learning skills to disclose abuse					0.053 (1, 156)	0.818	0.179***	0.148***
No (RC)	12	24	0.544 (0.249, 0.839)***					
Yes	22	134	0.586 (0.384, 0.787)***	0.042 (−0.315, 0.399)				
(12) Increasing child’s self-esteem					2.689 (1, 156)	0.103	0.182***	0.118***
No (RC)	30	113	0.528 (0.363, 0.693)***					
Yes	4	45	0.924 (0.477, 1.370)***	0.395 (−0.081, 0.871)				
Delivery techniques								
(1) Discussion/debate					0.050 (1, 156)	0.823	0.178***	0.149***
No (RC)	12	54	0.547 (0.273, 0.822)***					
Yes	22	104	0.587 (0.377, 0.796)***	0.039 (−0.306, 0.385)				
(2) Behavioral skills training techniques					2.322 (1, 156)	0.130	0.178***	0.135***
No (RC)	10	27	0.377 (0.075, 0.678)*					
Yes	24	131	0.652 (0.462, 0.842)***	0.275 (−0.081, 0.632)				
(3) Photos/pictures					1.520 (1, 156)	0.220	0.178***	0.142***
No (RC)	21	50	0.483 (0.265, 0.701)***					
Yes	13	108	0.689 (0.441, 0.937)***	0.206 (−0.124, 0.536)				
(4) Video					0.045 (1, 156)	0.833	0.178***	0.149***
No (RC)	22	120	0.584 (0.383, 0.786)***					
Yes	12	38	0.546 (0.250, 0.842)***	−0.038 (−0.396, 0.319)				
(5) Puppets					7.354 (1, 156)	0.007**	0.182***	0.100***
No (RC)	29	113	0.500 (0.341, 0.658)***					
Yes	5	45	1.096 (0.691, 1.500)***	0.596 (0.162, 1.031)**				
(6) Vignettes/stories					1.248 (1, 156)	0.266	0.179***	0.137***
No (RC)	20	65	0.489 (0.268, 0.709)***					
Yes	14	93	0.673 (0.434, 0.912)***	0.184 (−0.141, 0.509)				
(7) Workbook					0.006 (1, 156)	0.939	0.178***	0.151***
No (RC)	25	118	0.576 (0.379, 0.773)***					
Yes	9	40	0.562 (0.245, 0.878)***	−0.014 (−0.387, 0.358)				
(8) Modeling					0.001 (1, 156)	0.977	0.178***	0.150***
No (RC)	24	117	0.574 (0.376, 0.772)***					
Yes	10	41	0.568 (0.258, 0.878)**	−0.005 (−0.373, 0.363)				
(9) Games/quizzes					5.462 (1, 156)	0.021*	0.179***	0.110***
No (RC)	29	91	0.494 (0.328, 0.661)***					
Yes	5	67	0.966 (0.604, 1.328)***	0.472 (0.073, 0.870)*				
(10) Theater play					0.842 (1, 156)	0.360	0.179***	0.140***
No (RC)	29	147	0.596 (0.425, 0.767)***					
Yes	5	11	0.410 (−0.215, 0.588)	−0.187 (−0.588, 0.215)				

# Studies = number of studies; # ES = number of effect sizes; mean *d* = mean effect size Cohen’s *d*; CI = confidence interval; β_1_ = estimated regression coefficient; df = degrees of freedom; Level 2 variance = variance of effect sizes within studies; Level 3 variance = variance between studies

*p < .05; **p < .01; ***p < .001

^a^Omnibus test of al regression coefficients of the model

^b^*p* value of the omnibus test

^c^Including studies conducted in Ecuador and Nigeria

^+^*p* < .1

#### Study Characteristics

None of the coded study characteristics, including several sample, design, and outcome characteristics, significantly moderated the overall effect of school-based programs on child abuse-related knowledge.

#### Program Characteristics

The program duration and the number of program sessions significantly moderated the overall effect. Higher effects were found when the school-based programs lasted longer and comprised more sessions.

Several program components and techniques moderated the overall effect. We found larger effects for school-based programs with a focus on improving social–emotional skills of children (*d* = 0.909 versus *d* = 0.489) and teaching children to avoid self-blame (*d* = 0.776 versus *d* = 0.412). Larger effects were also found for school-based programs using puppets (*d* = 1.096 versus *d* = 0.500) and when games or quizzes were played (*d* = 0.966 versus *d* = 0.494).

### Moderator Analyses for Self-Protection Skills

Table 3 shows the results of the moderator analyses for the outcome self-protection skills.

**Table 3:** Results of the moderator analyses for skills

Moderator variables	# Studies	# ES	Intercept/mean *d* (95% BI)	β_1_(95% CI)	*F* (df1, df2)^a^	*p*^b^	Level 2 variance	Level 3 variance
Overall Effect	22	99	0.528 (0.262, 0.794)***			< 0.001***	0.121***	0.322***
Study characteristics								
Publication year	22	99	0.529 (0.260, 0.798)***	0.008 (−0.015, 0.032)	0.482 (1, 97)	0.489	0.122***	0.329***
Country					1.346 (3, 94)	0.264	0.120***	0.314***
Europe (RC)	5	36	0.948 (0.416, 1.479)***					
North America	11	35	0.373 (−0.007, 0.753)+	−0.575 (−1.228, 0.079)				
Australia	1	14	−0.019 (−1.149, 1.111)	−0.966 (−2.251, 0.283)				
Asia	4	14	0.533 (−0.029, 1.096)+	−0.414 (−1.188, 0.360)				
Sample characteristics								
Sample size	22	99	0.523 (0.246, 0.800)***	0.000 (−0.000, 0.001)	0.033 (1, 97)	0.856	0.122***	0.335***
Mean age of child (start study)	20	92	0.579 (0.314, 0.845)***	−0.123 (−0.231, −0.015)*	5.120 (1, 90)	0.026*	0.129***	0.276***
Type of school					22.319 (1, 97)	< 0.001***	.123***	.126***
Elementary school (RC)	19	81	0.326 (0.123, 0.528)**					
Preschool/kindergarten	3	18	1.529 (1.066, 1.992)***	1.203 (0.668, 1.709)***				
Mean age of parents (start study)	7	47	0.515 (−0.206, 1.237)	−0.197 (−0.540, 0.146)	1.340 (1, 45)	0.253	0.101***	0.818***
Percentage girls	22	99	0.470 (0.238, 0.702)***	−7.403 (−12.540, −2.266)**	8.181 (1. 97)	0.005**	0.122***	0.220***
Percentage non-Caucasians/non-whites	13	44	0.431 (0.190, 0.672)***	0.294 (−0.534, 1.122)	0.512 (1, 42)	0.478	0.060***	0.138***
Design characteristics								
Type of experiment					0.389 (2, 96)	0.679	0.122***	0.343***
Quasi-experimental (RC)	11	55	0.467 (0.085, 0.850)*					
RCT	4	18	0.396 (−0.253, 1.044)	−0.072 (−0.824, 0.681)				
Cluster RCT	7	26	0.706 (0.214, 1.197)**	0.238 (−0.385, 0.861)				
Intent-to-treat analysis					0.010 (1, 97)	0.920	0.121***	0.340***
No/unknown (RC)	14	63	0.518 (0.180, 0.856)**					
Yes	8	36	0.547 (0.087, 1.008)*	0.029 (−0.542, 0.600)				
Program fidelity					0.467 (1, 97)	0.496	0.121***	0.331***
Not reported/monitored (RC)	15	66	0.593 (0.265, 0.921)***					
Monitored and measured	7	33	0.394 (−0.078, 0.867)	−0.198 (−0.774, 0.377)				
Type of program in control group					1.594 (1, 91)	0.210	0.113***	0.158***
Waiting list (RC)	16	62	0.527 (0.273, 0.781)***					
No program	6	31	0.240 (−0.134 0.614)	−0.287 (−0.739, 0.165)				
Group differences at baseline					0.005 (1, 97)	0.942	0.121***	0.340***
No (RC)	8	34	0.542 (0.091, 0.992)*					
Yes	14	65	0.521 (0.178, 0.863)**	−0.021 (−0.587, 0.545)				
Outcome characteristics								
Outcome					1.659 (1, 97)	0.201	0.122***	0.305***
Self-protection skills (RC)	17	79	0.560 (0.295, 0.825)***					
Disclosure	5	20	0.381 (0.037, 0.726)*	−0.178 (−0.453, 0.096)				
Assessment type					3.860 (2, 94)	0.024*	0.110***	0.340***
Questionnaire (RC)	7	35	0.276 (−0.070, 0.621)					
Vignettes	14	54	0.669 (0.370, 0.968)***	0.393 (0.096, 0.691)*				
In-vivo simulation	1	8	0.489 (0.018, 0.960)*	0.214 (−0.263, 0.691)				
Follow-up period (in months)	7	29	0.320 (0.101, 0.539)**	−0.163 (−0.277, −0.048)**	8.482 (1, 27)	0.007**	0.049***	0.054*
Program characteristics								
Type of abuse					0.498 (1, 97)	0.482	0.121***	0.331***
Only sexual child abuse (RC)	18	93	0.571 (0.276, 0.867)***					
Any form of child abuse	4	6	0.315 (−0.341, 0.972)	−0.256 (−0.976, 0.464)				
Type of instructor					1.249 (2, 96)	0.292	0.121***	0.318***
Teacher (RC)	11	54	0.742 (0.357, 1.127)***					
External	9	28	0.383 (−0.039, 0.806)+	−0.359 (−0.930, 0.213)				
Combination	3	17	0.198 (−0.524, 0.921)	−0.543 (−1.362, 0.275)				
Training school personnel					5.333 (1, 69)	0.024*	.152***	.264***
No/unknown (RC)	4	16	1.213 (0.605, 1.821)***					
Yes	9	55	0.383 (0.005, 0.762)*	−0.829 (−1.546, −0.113)*				
Are parents involved?					4.973 (1, 97)	0.028*	0.121***	0.262***
No (RC)	15	74	0.340 (0.045, 0.636)*					
Yes	7	25	0.932 (0.496, 1.368)***	0.592 (0.065, 1.119)*				
Program duration (in weeks)	20	85	0.523 (0.229, 0.817)***	−0.077 (−0.215, 0.061)	1.241 (1, 83)	0.268	0.135***	0.332***
Number of sessions	21	97	0.583 (0.301, 0.865)***	0.071 (−0.030, 0.172)	1.927 (1, 95)	0.168	0.121***	0.329***
Duration of sessions (in minutes)	20	81	0.593 (0.342, 0.844)***	−0.017 (0.029, −0.005)**	8.159 (1, 79)	0.005**	0.144***	0.229***
Intensity sessions					2.699 (2, 82)	0.073+	0.131***	0.280***
Weekly (RC)	8	40	0.228 (−0.186, 0.642)					
More than once a week	7	37	0.937 (0.492, 1.382)***	0.709 (0.102, 1.317)*				
Only one session	5	8	0.565 (−0.006, 1.137)+	0.337 (−0.368, 1.043)				
Program components								
(1) Knowledge concepts/definitions					0.840 (1, 97)	0.362	.121***	.325***
No (RC)	15	79	0.608 (0.290, 0.926)***					
Yes	7	20	0.337 (−0.156, 0.830)	−0.271 (−0.858, 0.316)				
(2) Identifying trust person					4.792 (1, 97)	0.031*	0.121***	0.262***
No (RC)	15	64	0.717 (0.418, 1.016)***					
Yes	7	35	0.143 (−0.283, 0.569)	−0.574 (−1.095, −0.054)*				
(3) Learning about secrets					1.073 (1, 97)	0.303	0.121***	0.323***
No (RC)	13	48	0.647 (0.296, 0.998)***					
Yes	9	51	0.365 (−0.045, 0.776)+	−0.282 (−0.822, 0.258)				
(4) Increasing awareness of personal rights					0.903 (1, 97)	0.344	0.121***	0.321***
No (RC)	17	79	0.594 (0.295, 0.894)***					
Yes	5	20	0.283 (−0.294, 0.860)	−0.311 (−0.962, 0.339)				
(5) Increasing social–emotional skills					0.068 (1, 97)	0.794	0.121***	0.339***
No (RC)	18	93	0.512 (0.213, 0.811)***					
Yes	4	6	0.607 (−0.048, 1.262)+	0.095 (−0.625, 0.815)				
(6) Teaching to avoid self-blame					2.001 (1, 97)	0.160	0.122***	0.299***
No (RC)	13	63	0.370 (0.030, 0.710)*					
Yes	9	36	0.743 (0.345 1.141)***	0.373 (−0.150, 0.897)				
(7) Learning about own body and boundaries					0.025 (1, 97)	0.875	0.122***	0.338***
No (RC)	3	6	0.585 (−0.180, 1.351)					
Yes	19	93	0.520 (0.229, 0.811)***	−0.065 (−0.884, 0.753)				
(9) Recognizing and avoid risky situations					0.487 (1, 97)	0.487	0.121***	0.331***
No (RC)	12	45	0.616 (0.249, 0.982)**					
Yes	10	54	0.426 (0.028, 0.823)*	−0.190 (−0.731 0.351)				
(10) Increasing assertiveness skills					0.000 (1, 97)	0.996	0.121***	0.341***
No (RC)	6	33	0.527 (0.010, 1.045)*					
Yes	16	66	0.529 (0.208, 0.850)**	0.002 (−0.607, 0.611)				
(11) Learning to go away or to find help					0.045 (1, 97)	0.832	0.122***	0.338***
No (RC)	8	32	0.488 (0.025, 0.951)*					
Yes	14	67	0.549 (0.214, 0.885)**	0.061 (−0.511, 0.633)				
(12) Learning skills to disclose abuse					1.226 (1, 97)	0.271	0.121***	0.315***
No (RC)	7	42	0.319 (−0.140, 0.778)					
Yes	15	57	0.632 (0.309, 0.954)***	0.313 (−0.248, 0.874)				
(13) Increasing child’s self-esteem					3.842 (1, 97)	0.053+	0.125***	0.260***
No (RC)	20	94	0.453 (0.197, 0.709)***					
Yes	2	5	1.316 (0.480, 2.152)**	0.863 (−0.011, 1.737)+				
Delivery techniques								
(1) Discussion/debate					3.672 (1, 97)	0.058+	0.123***	0.268***
No (RC)	7	39	0.862 (0.436, 1.288)***					
Yes	15	60	0.357 (0.053, 0.661)*	−0.505 (−1.029, 0.018)+				
(2) Behavioral skills training techniques					0.318 (1, 97)	0.574	0.121***	0.333***
No (RC)	6	33	0.403 (−0.113, 0.919)					
Yes	16	66	0.575 (0.258, 0.892)***	0.172 (−0.434, 0.778)				
(3) Photos/pictures					1.617 (1, 97)	0.207	0.122***	0.307***
No (RC)	13	60	0.384 (0.039, 0.729)*					
Yes	9	39	0.723 (0.322 1.123)***	0.339 (−0.190, 0.867)				
(4) Video					0.265 (1, 97)	0.608	0.122***	0.330***
No (RC)	13	73	0.578 (0.248, 0.908)***					
Yes	10	26	0.458 (0.077, 0.840)*	−0.120 (−0.581, 0.342)				
(6) Vignettes/stories					3.250 (1, 97)	0.075+	0.122***	0.278***
No (RC)	12	57	0.314 (−0.031, 0.658)+					
Yes	10	42	0.770 (0.404, 1.137)***	0.457 (−0.046, 0.960)+				
(7) Workbook					0.034 (1, 97)	0.854	0.121***	0.339***
No (RC)	17	77	0.514 (0.203, 0.825)					
Yes	5	22	0.574 (0.010, 1.139)*	0.060 (−0.584, 0.704)				
(8) Modeling					0.040 (1, 97)	0.841	0.121***	0.341***
No (RC)	14	66	0.549 (0.210, 0.888)**					
Yes	8	33	0.491 (0.033, 0.950)*	−0.058 (−0.628, 0.513)				
(9) Games/quizzes					0.055 (1, 97)	0.815	0.122***	0.338***
No (RC)	20	96	0.538 (0.254, 0.822)***					
Yes	2	3	0.422 (−0.519, 1.362)	−0.116 (−1.099, 0.866)				
(10) Theater play					0.342 (1, 97)	0.560	0.122***	0.328***
No (RC)	18	87	0.546 (0.271, 0.821)***					
Yes	4	12	0.433 (0.013, 0.853)*	−0.113 (−0.495, 0.269)				

# Studies = number of studies; # ES = number of effect sizes; mean *d* = mean effect size Cohen’s *d*; CI = confidence interval; β_1_ = estimated regression coefficient; df = degrees of freedom; Level 2 variance = variance of effect sizes within studies; Level 3 variance = variance between studies

*p < .05; **p < .01; ***p < .001

^a^Omnibus test of al regression coefficients of the model

^b^*p* value of the omnibus test

^+^*p* < .1

#### Study Characteristics

A significant moderating effect for the mean age of children at the start of the study was found. Study samples with younger children yielded larger effect sizes. Further, we found larger effects for studies carried out in preschools or kindergarten (*d* = 1.531) than studies carried out in elementary schools (*d* = 0.329). The percentage of girls in the sample significantly moderated the overall effect. The effect sizes increased as the percentage of girls in the sample decreased. We also found that the use of vignettes (*d* = 0.669) produced larger effects than using questionnaires (*d* = 0.276). Finally, we found that effect sizes increased as the follow-up duration decreased.

#### Program Characteristics

Larger effects were found when school-based programs were delivered by school personnel without any training (*d* = 1.213) compared to programs provided by trained school personnel (*d* = 0.383). A significant moderating effect was also found for the involvement of parents. School-based programs involving parents yielded larger effects (*d* = 0.932) than programs not involving parents (*d* = 0.340). Larger effects were also found for programs with shorter sessions. For the program components, we found significantly smaller effects for programs focusing on identifying a trusted person (*d* = 0.143 versus *d* = 0.717). For the delivery techniques, no significant moderating effects were found.

## Discussion

This study was aimed at gaining insight into the effect of school-based child abuse prevention programs on child abuse-related knowledge and self-protection skills. Furthermore, we aimed to explore the program components, delivery techniques, and other study and program characteristics that were assumed to be associated with this effectiveness. In pursuing these aims, we conducted two three-level meta-analyses in which the overall effects of school-based programs on child abuse-related knowledge and children’s self-protection skills were examined, and in which the moderating effects of sample, study, and program characteristics were tested.

We found a significant overall effect of *d* = 0.572 of school-based prevention programs on child abuse-related knowledge, and a significant overall effect of *d* = 0.528 on children’s self-protections skills. These effects were medium in magnitude and in line with findings of previously conducted meta-analyses showing medium-to-large effects on knowledge and skills of school-based programs for child sexual abuse prevention (Davis & Gidycz, 2000; Rispens et al., 1997; Walsh et al., 2018). The results of the trim-and-fill analyses suggest that in particular above-average effect sizes may have been missing in both meta-analyses. This implies that there were no indications for publication bias as studies producing non-significant and/or negative results seemed sufficiently represented. It must be stressed that the performance of the trim-and fill method is limited in 3-level meta-analytic models, as this bias assessment method assumes effect size independency and homogeneity in effect sizes (Nakagawa & Santos, 2012; Terrin et al., 2003). Therefore, the results of the bias assessment must be interpreted with caution.

In this meta-analytic review, disclosing child abuse during or after the program or outcomes related to disclosure (i.e., disclosure intentions or having confidence to disclose) were considered as protective behaviors. These outcomes were examined in the meta-analysis on self-protection skills and therefore, the overall effect of school-based prevention programs on self-protection skills partially represents the effect of these programs on abuse disclosure. To examine differences in program effects between disclosure outcomes and self-protection skills, the type of outcome was tested as a moderator (see Table 3). Although no significant moderating effect was found, we did find a significant mean effect for disclosure outcomes (*d* = 0.381) and for self-protection skills (*d* = 0.560). This indicates that school-based child abuse prevention programs may very well be effective in increasing both self-protection skills and child abuse disclosure. As for the latter, this aligns with the previous findings stating that children participating in school-based prevention programs are more likely to disclose abuse to their teacher or other adults they trust (MacIntyre & Carr, 1999; Topping & Barron, 2009). However, only two of the included primary studies reported on actual disclosures of children during or after the school prevention program. Future research should therefore measure abuse disclosures of children.

### The Moderating Effect of Study Characteristics

For the self-protection skills, we found larger effects in samples of younger children and in preschool or kindergarten samples than in elementary school children. These findings underline the importance of applying a preventive approach to child abuse. Rispens et al. (1997) as well as Davis and Gidycz (2000) also found that younger children benefit more from school-based child abuse prevention programs than older children. Davis and Gidycz (2000) suggest that smaller effect sizes for older age groups could be the result of ceiling effects, arguing that older children may have already adopted some abuse-related knowledge or skills and could hardly perform better on the assessed outcomes. Rispens et al. (1997) found that age group differences disappeared at follow-up evaluations and discussed that younger children may not retain the learned information as well as older children.

Larger positive effects were also found in samples with less girls. This indicates that boys may benefit more from the school-based programs in terms of improving their self-protection skills. This is not in line with the previous studies, which indicated that boys benefit less from school-based prevention programs due to potential traditional stereotypes positing men as the aggressors rather than victims of abuse, creating barriers to boys’ successful engagement in child abuse prevention programs (Nickerson et al., 2019; Scholes et al., 2014a, 2014b). Previous research also suggests that boys were more reluctant to disclose child abuse compared to girls (Gagnier & Collin-Vézina, 2016; Lev-Wiesel & First, 2018), which is also not in line with our findings, as child abuse disclosure was regarded as a protective behavior in our meta-analysis. However, it should be noted that other studies did not find gender differences in the effect of school-based programs (Tutty, 1997; Wurtele & Owens, 1997), suggesting inconsistency in previous research on the influence of gender on the effectiveness of these programs.

Larger effect sizes were found for studies using vignettes to measure self-protection skills compared to studies using questionnaires. Not in line with this finding, Davis and Gidycz (2000) found larger effects for studies using behavioral observations to measure self-protection skills in their meta-analytic evaluation, such as in-vivo simulations. In another meta-analysis, Walsh et al. (2018) exclusively included studies using in-vivo simulations to assess children’s self-protection skills in responding to actual threats, as such simulations may be the most direct method of measuring behavioral change. However, both meta-analyses only included two studies using simulations and therefore their findings should be interpreted with caution. It was also argued that simulating abusive situations raises important ethical concerns (Boyle & Lutzker, 2005; Kenny et al., 2008; Walsh et al., 2018). Vignettes presented in different ways (e.g., written narrative, videotape, audiotape) might be less ethically challenging and easier for researchers, and are therefore widely used (Topping & Barron, 2009). Using vignettes as a measure of self-protection skills may therefore be considered in future research. However, the potential of vignettes in measuring behavioral change is questionable, as vignettes may rather measure the ability to recommend an appropriate behavioral response to an abusive situation (see the “Limitations” section).

Finally, smaller program effects on self-protection skills were found when follow-up durations increased. This is in line with the findings of Rispens et al. (1997) who found smaller—but still significant and substantial—follow-up effects compared to post-intervention effects. They indicated that this might be due to fading program effects. *Fade-out effects* refer to circumstances in which the outcomes of an experimental manipulation diminish after a particular intervention has ended (Sutherland et al., 2017). Fade-out can be complete or partial. Complete fade-out is where intervention effects reduce to zero, whereas partial fade-out represents a reduction in the magnitude of the effect over time but still with a discernable difference between treatment and control participants. Fade-out effects are common in interventions targeting children’s cognitive, social, or emotional development (Bailey et al., 2017). In the context of improving children’s self-protections skills, fade-out effects could be due to children not being able to generalize their acquired skills from simulated to actual settings. School-based programs may benefit from extended time periods or booster sessions so that more opportunities for practicing skills can be provided to the children (Kenny et al., 2008; MacIntyre & Carr, 2000; Rispens et al., 1997).

### The Moderating Effect of Program Characteristics

For child abuse-related knowledge, we found that the program duration and number of sessions significantly moderated the overall effect of school-based programs. School-based programs with a longer duration and more sessions did have significantly larger effects on child abuse-related knowledge, indicating that knowledge gains can be achieved by increasing the time spent learning about prevention and child abuse. These results are consistent with those of the previous review studies on the effect of school-based child abuse prevention programs (Davis & Gidycz, 2000; Kenny et al., 2008; MacIntyre & Carr, 2000; Rispens et al., 1997; Topping & Barron, 2009).

We also found several program characteristics moderating the effect of school-based programs on self-protections skills. First, we found that programs delivered by school personnel without any training showed larger effects than programs delivered by trained school personnel. This finding was surprising and not in line with the previous research indicating that school-based programs taught by trained instructors were most effective in increasing safety skills (MacIntyre & Carr, 2000). Taking a closer look at the programs delivered by untrained school personnel which were examined in the four studies, we found that two of these studies reported on the effect of the Body Safety Training Program: Teacher Version (BST; Wurtele, 1986) and produced 14 out of the 16 effect sizes. The lessons of this particular program are led by classroom teachers who read from an extensive script. Throughout the script there are prompts to ask children questions, practice skills, or use language of encouragement. Although teachers do not receive any training in the core concepts of the program, they are guided and supported by a precise script that prevents teachers from modifying the content of the program so that it is aligned with their own personal beliefs. In this way, program fidelity is assured. Furthermore, it should be noted that studies in which no information was provided about whether or not school personnel was trained were coded as untrained. However, school personnel could have been trained in reality. This should be taken into account in interpreting this result.

Second, we found larger effects for school-based programs actively involving the parents of the participating children, for example, by encouraging parents to practice safety skills at home with the children. In accordance with this finding, previous review studies also refer to the importance of parental involvement in school-based programs and indicate that involving parents helped to improve and maintain self-protection skills (Kenny et al. 2008; MacIntyre & Carr, 2000; Topping & Barron, 2009). Research suggests that preschool children are more likely to learn skills when these are introduced by their parents and when children have the opportunity to repeatedly rehearse the skill during role-plays in different settings (Boyle & Lutzker, 2005; Deblinger et al., 2001). Finally, larger effect sizes were found for school-based prevention programs with shorter sessions. This was in line with the findings of Davis and Gidycz (2000). They argue that programs in which the content is divided into shorter segments allow children to maintain their attention for the entire period and increases the amount of repetition of the material, thus leading to greater retention of the material.

### Effective Program Components and Techniques

We found several program components and delivery techniques that were associated with the effectiveness of school-based child abuse prevention programs. For child abuse-related knowledge, larger effect sizes were found for school-based programs focusing on increasing the social–emotional skills of children and teaching children to avoid self-blame. The social–emotional skills component included teaching children skills regarding social relationships, empathy, emotion management, and problem-solving. Previous research indicated that it is beneficial for abuse prevention programs to address training in skill development in positive areas, such as problem-solving and communication (Daro, 1994). Better social–emotional skills enable children to empathize with others who are in abusive situations, which may increase their knowledge on abuse concepts, such as the difference between appropriate and inappropriate touch, or between safe and unsafe situations. Research also indicates that children with better social problem-solving skills are more adept at resolving child abuse-related dilemmas, because they offer more thoughtful solutions to different situations (Grober & Bogat, 1994; Sanderson, 2004). Therefore, children might benefit more from prevention education focusing on social problem-solving.

Teaching children that abuse is never the child’s fault was also associated with larger effects of school-based programs on knowledge. Child maltreatment often promotes a self-blaming or pessimistic attributional style, possibly due to internalizing parental (or others who inflict maltreatment) negative beliefs about them (Carlson et al., 1997; Messman-Moore & Coates, 2007). In accordance with our findings, Kenny et al. (2008) suggested in their review that components of successful prevention programs included reassuring children that abuse is not their fault. It should be noted that learning that child abuse is never the child’s fault was to some extent incorporated into the outcome abuse-related knowledge, as some of the outcome measures for knowledge covered questions about self-blame. This may have influenced our findings.

For child abuse-related knowledge, larger effects were found for school-based programs using puppets as a delivery technique. Puppets are often used in school-based programs to serve as models with which children can identify at both affective and cognitive levels (Dhooper & Schneider, 1995). In line with our findings, preliminary research indicates that use of puppets can increase the effectiveness of school-based programs (Davis & Gidycz, 2000). It should, however, be noted that in our meta-analysis large and significant correlations were found between the variable indicating the use of puppets and other components, including the social–emotional skill component (*r* = 0.76) and the self-esteem component (*r* = 0.85). This implies multicollinearity (see the “Limitations” section) and indicates that programs often use puppets as well as focus on increasing the child’s social–emotional skills or self-esteem. Therefore, it is not entirely clear which of these two components actually lead to a greater effect.

We found a significant moderating effect of games as a delivery technique in school-based programs for increasing abuse-related knowledge. Overall, games have a positive impact on students learning as they increase their motivation, confidence, effort, and involvement in their learning, and are therefore often used in a child abuse prevention context to increase children’s knowledge and skills (Scholes, et al., 2014a, 2014b). In line with our findings, previous research indicates that school-based prevention programs that encourage active participation of children, for example through games and role-plays, are more effective than those that use either passive methods (e.g., traditional teaching, discussions) or no participation (e.g., videos, written materials; Davis & Gidycz, 2000).

Finally, for self-protection skills, significantly smaller effect sizes were found for school programs focusing on identifying a trusted person for a child. This finding was unexpected as school-based programs for the prevention of child abuse often include identifying adults who can be trusted and encourage children to tell these trusted adults in the case of a potential abuse situation (Citak Tunc et al., 2018).

## Limitations

Several limitations need to be discussed. First, we aimed to examine the effectiveness of school-based prevention programs on any form of child abuse and identify potential effective components. Although we focused on a broad definition of child abuse, most of the included school-based prevention programs are focused on the prevention of child sexual abuse either or not in combination with other forms of abuse. To our knowledge, there are no primary studies on school-based programs for non-sexual child abuse. There may be differences in program components and techniques that are associated with program effectiveness between programs targeting sexual abuse only and programs targeting any form of abuse. Unfortunately, the number of studies reporting on programs targeting any form of abuse that could be included in the current review (*k* = 8) did not allow for conducting intelligible analyses to examine these differences. To increase our knowledge on what components and techniques may best support programs targeting non-sexual abuse, it is necessary that the effectiveness of these programs is examined in future primary research.

Second, another shortcoming of the primary studies included in this meta-analysis is that they did not report on whether child abuse prevention was actually achieved. All studies evaluated program effectiveness in terms of knowledge and skills gains, and therefore we used these outcome measures in our meta-analyses. However, this does not directly indicate that program participants are less likely to experience child abuse. Future research should focus on this, for example, by comparing (self-reported) child abuse rates in a (quasi-) experimental design. An important challenge in this design is the need for a very large sample, as the prevalence of child abuse in the general population is relatively low. Further, asking children to report incidents of child abuse victimization could be a great burden for them. Properly validating the preventive effects of school-based programs is therefore challenging, both from a practical and ethical point of view.

A third limitation is related to the way self-protection skills were measured. We included studies measuring self-protection skills with questionnaires, vignettes, and in-vivo simulations. It is argued that in-vivo simulation techniques are the most direct way to assess children’s skills in responding to actual threats (Boyle & Lutzker, 2005; Walsh et al., 2018). Vignettes or questionnaires are presented as abstract situations and possibilities. These measures might therefore not evaluate how an individual will actually respond in a potentially abusive situation (Kenny et al., 2008; Tutty, 1997), but rather evaluate the ability to recommend an appropriate behavioral response. However, using in-vivo simulations to measure children’s responses to potential abusers raises important ethical issues (Boyle & Lutzker, 2005). It can also not be assumed that behavioral responses will be generalized from simulated to actual settings in the context of an approach from an unknown adult toward a child in a school hallway or playground (Walsh et al., 2018). These considerations should be taken into account in future research.

Fourth, large correlation coefficients were found between the variables that we included in the moderator analyses. For the outcome child abuse-related knowledge, 16% of the correlations were significant and large in magnitude, according to Cohen (1988; larger than *r* = 0.5 or smaller than *r* = − 0.5; the correlation tables are available from the corresponding author on request). This was 13% for the outcome self-protection skills. This indicates that there is multicollinearity in our data, meaning that one or more predictors in a regression model can be (linearly) predicted by another predictor with relatively high accuracy (Harrell, 2015). This should be taken into account in interpreting our results. Because of this it was not possible to examine the unique contribution of the significant moderators in a multiple moderator model, as the absence of multicollinearity is an assumption for such model.

Finally, some categories of the variables tested in the moderator analyses were based on only one or two studies. In interpreting both significant and non-significant results of these moderator analyses, the small cell sizes limit drawing firm conclusions and call the results of these analyses into question. The findings do represent a call for future research, for example, on the effect of school-based programs in high schools and on the effect of in-vivo simulation methods on self-protection skills.

## Implications for Clinical Practice and Future Research

This study provides important knowledge for clinical practice and suggestions for future research. We found positive significant effects of school-based child abuse prevention programs on increasing the knowledge of children about child abuse or prevention as well as on children’s self-protection skills. Although school-based programs for child (sexual) abuse prevention are widely adopted across the Unites States (Finkelhor et al., 1995; Gibson & Leitenberg, 2000), there is less attention for school-based child abuse education in European countries (World Health Organization, 2018). Our positive results suggest that child abuse prevention or education programs should be included as a standard part of the curriculum in primary and secondary education. It is also important to implement these programs in an early stage, as we found large effects for young children and for programs provided in preschool or kindergarten. Furthermore, we found several program components and techniques that were associated with greater effectiveness of school-based programs. Programs focusing on strengthening social–emotional skills of children, avoiding self-blame, using puppets, and using games or quizzes yielded larger effects on child abuse-related knowledge. Program effectiveness could possibly be improved by integrating these components and techniques into these programs. These results can also be used in the development of new and promising school-based programs in which the most effective components should be integrate.

As mentioned before, we found that most of the primary studies we included focused on school-based programs for the prevention of sexual abuse, while in fact this is the least prevalent form of child abuse (Stoltenborgh et al., 2015). Therefore, it is important that existing or newly developed school programs widen their scope to the prevention of other forms of child abuse, such as physical abuse, emotional abuse, and neglect. Future experimental research should focus on examining the effectiveness of school-based prevention programs for these forms.

Finally, given the substantial changes to school settings in the current COVID-19 pandemic, one can ask how the findings of this review relate to these changed settings. To combat the spread of COVID-19, many primary and secondary schools were closed and in-person classes were replaced with remote instruction and hybrid learning. If offered at all, it is likely that school-based child abuse prevention programs were adapted to an online learning environment, which probably influences the effectiveness of these programs. Since there is evidence that incidences of child abuse and neglect has increased during the COVID-19 pandemic (i.e., Agrawal, 2020; Kovler et al., 2020), it is important that programs as examined in this review are continued to be offered in some form or another in order to fight increasing child abuse rates. Certain elements of school-based child abuse prevention programs are potentially suitable to be converted from an in-person context to a web-based or mobile-based context, such as learning about the concept of child abuse, safe or unsafe situations, and self-assertiveness skills. These topics were also covered by a child sexual abuse prevention education program using a hybrid application, developed by Kang et al. (2020). This online program showed positive effects for elementary school students, especially for self-protective behaviors, and can therefore be a good alternative for child abuse prevention programs in a school context (Kang et al., 2020).

## Conclusion

Our findings show that school-based child abuse prevention programs are effective in increasing child abuse-related knowledge and self-protections skills of children. As for the former outcome, we found that programs seem more effective when programs had a longer duration and more sessions. For self-protection skills, we found larger program effects for young children, preschool or kindergarten children, boys, and when skills were measured with vignettes. Larger program effects were also found for programs providing no training for school personnel, programs involving parents, and programs with short sessions. Moreover, we found that several program components and techniques were associated with greater program effectiveness, including increasing social–emotional skills of children, avoiding self-blame, using puppets, and using games or quizzes. Our findings can be used to improve existing school programs, for example, by integrating effective components in programs, or by developing new promising school-based programs that comprise the most effective components.

## Supplementary Information

Below is the link to the electronic supplementary material.

Supplementary file1 (DOCX 120 KB)
