# Supplementary material for: Effective Components of School-Based Prevention Programs for Child Abuse: A Meta-Analytic Review
Source: Clin Child Fam Psychol Rev. 2021 Jun 4;24(3):553–78. doi: 10.1007/s10567-021-00353-5 (PMC8176877; doi:10.1007/s10567-021-00353-5)
Supplement: Supplementary file 1 — Supplementary file1 (DOCX 120 KB) [file 10567_2021_353_MOESM1_ESM.docx]

Appendix A

Search Syntax

The searches below yielded the following results on February 14^th^, 2020; PsycINFO (553 references), ERIC (229 references), Web of Science (449 references), and SocINDEX (211).

**PsycINFO (Ovid, 1806 to February 14th, 2020)**

1. child abuse/ OR child abuse reporting/ OR child neglect/ OR domestic violence/ OR emotional abuse/ OR incest/ OR physical abuse/ OR rape/ OR sexual abuse/ OR sexual harassment/ OR verbal abuse/ OR (child abus* OR child maltreat* OR child* welfare recidiv* OR neglect OR out-of-home placement* OR psychological abus* OR sexual abus* OR emotional abus* OR physical abus* OR verbal abus* OR domestic violen* OR incest OR rape OR sexual harass* OR sexual assault*).ti,ab,id,tm.
2. ((school* OR education*) ADJ3 (intervention* OR program* OR training* OR project* OR module) OR school education OR prevention program* OR protection).ti,ab,id.
3. kindergartens/ OR kindergarten students/ OR preschool education/ OR preschool students/ OR preschool teachers/ OR elementary education/ OR elementary schools/ OR elementary school students/ OR elementary school teachers/ OR primary school students/ OR public school education/ OR middle schools/ OR middle school education/ OR middle school students/ OR middle school teachers/ OR junior high schools/ OR junior high school students/ OR junior high school teachers/ OR secondary education/ OR high schools/ OR high school students/ OR high school education/ OR high school teachers/ OR (kindergarten* OR early childhood education OR preschool* OR elementary education OR elementary school* OR primary education OR primary school* OR K-12* OR K12 OR 1st-grade* OR first-grade* OR grade 1 OR grade one OR 2nd-grade* OR second-grade* OR grade 2 OR grade two OR 3rd-grade* OR third-grade* OR grade 3 OR grade three OR 4th-grade* OR fourth-grade* OR grade 4 OR grade four OR 5th-grade* OR fifth-grade* OR grade 5 OR grade five OR 6th-grade* OR sixth-grade* OR grade 6 OR grade six OR intermediate general OR middle school* OR secondary education OR secondary school* OR 7th-grade* OR seventh-grade* OR grade 7 OR grade seven OR 8th-grade* OR eight-grade* OR grade 8 OR grade eight OR 9th-grade* OR ninth-grade* OR grade 9 OR grade nine OR 10th-grade* OR tenth-grade* OR grade 10 OR grade ten OR 11th-grade* OR eleventh-grade* OR grade 11 OR grade eleven OR 12th-grade* OR twelfth-grade* OR grade 12 OR grade twelve OR junior high* OR highschool* OR high school*).ti,ab,id.
4. (random* OR RCT OR control group* OR comparison group* OR post-treatment OR pre-treatment OR pre-test* OR pretest* OR post-test* OR posttest* OR waitlist OR waitinglist OR wait* list OR intervention* OR program* OR training* OR project* OR child abuse potential inventor* OR capi OR conflict tactics scale* OR effect size* ).ti,ab,id. OR (child abuse potential inventor* OR capi OR conflict tactics scale*).tm.
5. 1 AND 2 AND 3 AND 4

Key: / = subject heading, ti = title, ab = abstract, id = key concepts (other keywords added by PsycINFO indexers to supplement the subject headings), tm = tests & measures.

**ERIC (Ovid, 1965 to February 2020)**

1. child abuse/ OR child neglect/ OR family violence/ OR rape/ OR sexual abuse/ OR sexual harassment/ OR (child abus* OR child maltreat* OR child* welfare recidiv* OR neglect OR out-of-home placement* OR psychological abus* OR sexual abus* OR emotional abus* OR physical abus* OR verbal abus* OR domestic violen* OR incest OR rape OR sexual harass* OR sexual assault*).ti,ab,id.
2. ((school* OR education*) ADJ3 (intervention* OR program* OR training* OR project* OR module) OR school education OR prevention program* OR protection).ti,ab,id.
3. (preschool education OR elementary secondary education OR grade 1 OR grade 2 OR grade 3 OR grade 4 OR grade 5 OR grade 6 OR grade 7 OR grade 8 OR grade 9 OR grade 10 OR grade 11 OR grade 12 OR elementary education OR primary education OR intermediate grades OR middle schools OR junior high schools OR secondary education OR high schools).el. OR kindergarten/ OR preschool education/ OR preschool teachers/ OR elementary secondary education/ OR grade 1/ OR grade 2/ OR grade 3/ OR grade 4/ OR grade 5/ OR grade 6/ OR grade 7/ OR grade 8/ OR grade 9/ OR grade 10/ OR grade 11/ OR grade 12/ OR elementary education/ OR elementary schools/ OR elementary school students/ OR elementary school teachers/ OR primary education/ OR public schools/ OR public school teachers/ OR middle schools/ OR middle school students/ OR middle school teachers/ OR junior high schools/ OR junior high school students/ OR secondary education/ OR secondary schools/ OR secondary school students/ OR secondary school teachers/ OR high schools/ OR high school students/ OR (kindergarten* OR early childhood education OR preschool* OR elementary education OR elementary school* OR primary education OR primary school* OR K-12* OR K12 OR 1st-grade* OR first-grade* OR grade 1 OR grade one OR 2nd-grade* OR second-grade* OR grade 2 OR grade two OR 3rd-grade* OR third-grade* OR grade 3 OR grade three OR 4th-grade* OR fourth-grade* OR grade 4 OR grade four OR 5th-grade* OR fifth-grade* OR grade 5 OR grade five OR 6th-grade* OR sixth-grade* OR grade 6 OR grade six OR intermediate general OR middle school* OR secondary education OR secondary school* OR 7th-grade* OR seventh-grade* OR grade 7 OR grade seven OR 8th-grade* OR eight-grade* OR grade 8 OR grade eight OR 9th-grade* OR ninth-grade* OR grade 9 OR grade nine OR 10th-grade* OR tenth-grade* OR grade 10 OR grade ten OR 11th-grade* OR eleventh-grade* OR grade 11 OR grade eleven OR 12th-grade* OR twelfth-grade* OR grade 12 OR grade twelve OR junior high* OR highschool* OR high school*).ti,ab,id.
4. (random* OR RCT OR control group* OR comparison group* OR post-treatment OR pre-treatment OR pre-test* OR pretest* OR post-test* OR posttest* OR waitlist OR waitinglist OR wait* list OR intervention* OR program* OR training* OR project* OR child abuse potential inventor* OR capi OR conflict tactics scale* OR effect size* ).ti,ab,id.
5. 1 AND 2 AND 3 AND 4
6. Limit 4 to peer review

**Web of Science (Thomson Reuters, Web of Science Core Collection, 1975 to 2020)**

1. TS=("child abus*" OR "child maltreat*" OR "child* welfare recidiv*" OR "neglect" OR "out-of-home placement*" OR "psychological abus*" OR "sexual abus*" OR "emotional abus*" OR "physical abus*" OR "verbal abus*" OR "domestic violen*" OR "incest" OR "rape" OR "sexual harass*" OR "sexual assault*")
2. TS=(("school*" OR "education*") NEAR/2 ("intervention*" OR "program*" OR "training*" OR "project*" OR "module") OR "school education" OR "prevention program*" OR "protection")
3. TS=("kindergarten*" OR "early childhood education" OR "preschool*" OR "elementary education" OR "elementary school*" OR "primary education" OR "primary school*" OR "K-12*" OR "K12" OR "1st-grade*" OR "first-grade*" OR "grade 1" OR "grade one" OR "2nd-grade*" OR "second-grade*" OR "grade 2" OR "grade two" OR "3rd-grade*" OR "third-grade*" OR "grade 3" OR "grade three" OR "4th-grade*" OR "fourth-grade*" OR "grade 4" OR "grade four" OR "5th-grade*" OR "fifth-grade*" OR "grade 5" OR "grade five" OR "6th-grade*" OR "sixth-grade*" OR "grade 6" OR "grade six" OR "intermediate general" OR "middle school*" OR "secondary education" OR "secondary school*" OR "7th-grade*" OR "seventh-grade*" OR "grade 7" OR "grade seven" OR "8th-grade*" OR "eight-grade*" OR "grade 8" OR "grade eight" OR "9th-grade*" OR "ninth-grade*" OR "grade 9" OR "grade nine" OR "10th-grade*" OR "tenth-grade*" OR "grade 10" OR "grade ten" OR "11th-grade*" OR "eleventh-grade*" OR "grade 11" OR "grade eleven" OR "12th-grade*" OR "twelfth-grade*" OR "grade 12" OR "grade twelve" OR "junior high*" OR "highschool*" OR "high school*")
4. TS=("random*" OR "RCT" OR "control group*" OR "comparison group*" OR "post-treatment" OR "pre-treatment" OR "pre-test*" OR "pretest*" OR "post-test*" OR "posttest*" OR "waitlist" OR "waitinglist" OR "wait* list" OR "intervention*" OR "program*" OR "training*" OR "project*" OR "child abuse potential inventor*" OR "capi" OR "conflict tactics scale*" OR "effect size*")
5. 1 AND 2 AND 3 AND 4

Key: TS = topic, which includes title, abstract, author keywords and Web of Science Keywords Plus

**SocINDEX (EBSCO, 1975 to 2020)**

1. TI("child abus*" OR "child maltreat*" OR "child* welfare recidiv*" OR "neglect" OR "out-of-home placement*" OR "psychological abus*" OR "sexual abus*" OR "emotional abus*" OR "physical abus*" OR "verbal abus*" OR "domestic violen*" OR "incest" OR "rape" OR "sexual harass*" OR "sexual assault*") OR AB("child abus*" OR "child maltreat*" OR "child* welfare recidiv*" OR "neglect" OR "out-of-home placement*" OR "psychological abus*" OR "sexual abus*" OR "emotional abus*" OR "physical abus*" OR "verbal abus*" OR "domestic violen*" OR "incest" OR "rape" OR "sexual harass*" OR "sexual assault*") OR KW("child abus*" OR "child maltreat*" OR "child* welfare recidiv*" OR "neglect" OR "out-of-home placement*" OR "psychological abus*" OR "sexual abus*" OR "emotional abus*" OR "physical abus*" OR "verbal abus*" OR "domestic violen*" OR "incest" OR "rape" OR "sexual harass*" OR "sexual assault*")
2. TI(("school*" OR "education*") NEAR/2 ("intervention*" OR "program*" OR "training*" OR "project*" OR "module") OR "school education" OR "prevention program*" OR "protection") OR AB(("school*" OR "education*") NEAR/2 ("intervention*" OR "program*" OR "training*" OR "project*" OR "module") OR "school education" OR "prevention program*" OR "protection") OR KW(("school*" OR "education*") NEAR/2 ("intervention*" OR "program*" OR "training*" OR "project*" OR "module") OR "school education" OR "prevention program*" OR "protection")
3. TI("kindergarten*" OR "early childhood education" OR "preschool*" OR "elementary education" OR "elementary school*" OR "primary education" OR "primary school*" OR "K-12*" OR "K12" OR "1st-grade*" OR "first-grade*" OR "grade 1" OR "grade one" OR "2nd-grade*" OR "second-grade*" OR "grade 2" OR "grade two" OR "3rd-grade*" OR "third-grade*" OR "grade 3" OR "grade three" OR "4th-grade*" OR "fourth-grade*" OR "grade 4" OR "grade four" OR "5th-grade*" OR "fifth-grade*" OR "grade 5" OR "grade five" OR "6th-grade*" OR "sixth-grade*" OR "grade 6" OR "grade six" OR "intermediate general" OR "middle school*" OR "secondary education" OR "secondary school*" OR "7th-grade*" OR "seventh-grade*" OR "grade 7" OR "grade seven" OR "8th-grade*" OR "eight-grade*" OR "grade 8" OR "grade eight" OR "9th-grade*" OR "ninth-grade*" OR "grade 9" OR "grade nine" OR "10th-grade*" OR "tenth-grade*" OR "grade 10" OR "grade ten" OR "11th-grade*" OR "eleventh-grade*" OR "grade 11" OR "grade eleven" OR "12th-grade*" OR "twelfth-grade*" OR "grade 12" OR "grade twelve" OR "junior high*" OR "highschool*" OR "high school*") OR AB("kindergarten*" OR "early childhood education" OR "preschool*" OR "elementary education" OR "elementary school*" OR "primary education" OR "primary school*" OR "K-12*" OR "K12" OR "1st-grade*" OR "first-grade*" OR "grade 1" OR "grade one" OR "2nd-grade*" OR "second-grade*" OR "grade 2" OR "grade two" OR "3rd-grade*" OR "third-grade*" OR "grade 3" OR "grade three" OR "4th-grade*" OR "fourth-grade*" OR "grade 4" OR "grade four" OR "5th-grade*" OR "fifth-grade*" OR "grade 5" OR "grade five" OR "6th-grade*" OR "sixth-grade*" OR "grade 6" OR "grade six" OR "intermediate general" OR "middle school*" OR "secondary education" OR "secondary school*" OR "7th-grade*" OR "seventh-grade*" OR "grade 7" OR "grade seven" OR "8th-grade*" OR "eight-grade*" OR "grade 8" OR "grade eight" OR "9th-grade*" OR "ninth-grade*" OR "grade 9" OR "grade nine" OR "10th-grade*" OR "tenth-grade*" OR "grade 10" OR "grade ten" OR "11th-grade*" OR "eleventh-grade*" OR "grade 11" OR "grade eleven" OR "12th-grade*" OR "twelfth-grade*" OR "grade 12" OR "grade twelve" OR "junior high*" OR "highschool*" OR "high school*") OR KW("kindergarten*" OR "early childhood education" OR "preschool*" OR "elementary education" OR "elementary school*" OR "primary education" OR "primary school*" OR "K-12*" OR "K12" OR "1st-grade*" OR "first-grade*" OR "grade 1" OR "grade one" OR "2nd-grade*" OR "second-grade*" OR "grade 2" OR "grade two" OR "3rd-grade*" OR "third-grade*" OR "grade 3" OR "grade three" OR "4th-grade*" OR "fourth-grade*" OR "grade 4" OR "grade four" OR "5th-grade*" OR "fifth-grade*" OR "grade 5" OR "grade five" OR "6th-grade*" OR "sixth-grade*" OR "grade 6" OR "grade six" OR "intermediate general" OR "middle school*" OR "secondary education" OR "secondary school*" OR "7th-grade*" OR "seventh-grade*" OR "grade 7" OR "grade seven" OR "8th-grade*" OR "eight-grade*" OR "grade 8" OR "grade eight" OR "9th-grade*" OR "ninth-grade*" OR "grade 9" OR "grade nine" OR "10th-grade*" OR "tenth-grade*" OR "grade 10" OR "grade ten" OR "11th-grade*" OR "eleventh-grade*" OR "grade 11" OR "grade eleven" OR "12th-grade*" OR "twelfth-grade*" OR "grade 12" OR "grade twelve" OR "junior high*" OR "highschool*" OR "high school*")
4. TI("random*" OR "RCT" OR "control group*" OR "comparison group*" OR "post-treatment" OR "pre-treatment" OR "pre-test*" OR "pretest*" OR "post-test*" OR "posttest*" OR "waitlist" OR "waitinglist" OR "wait* list" OR "intervention*" OR "program*" OR "training*" OR "project*" OR "child abuse potential inventor*" OR "capi" OR "conflict tactics scale*" OR "effect size*") OR AB("random*" OR "RCT" OR "control group*" OR "comparison group*" OR "post-treatment" OR "pre-treatment" OR "pre-test*" OR "pretest*" OR "post-test*" OR "posttest*" OR "waitlist" OR "waitinglist" OR "wait* list" OR "intervention*" OR "program*" OR "training*" OR "project*" OR "child abuse potential inventor*" OR "capi" OR "conflict tactics scale*" OR "effect size*") OR KW("random*" OR "RCT" OR "control group*" OR "comparison group*" OR "post-treatment" OR "pre-treatment" OR "pre-test*" OR "pretest*" OR "post-test*" OR "posttest*" OR "waitlist" OR "waitinglist" OR "wait* list" OR "intervention*" OR "program*" OR "training*" OR "project*" OR "child abuse potential inventor*" OR "capi" OR "conflict tactics scale*" OR "effect size*")
5. 1 AND 2 AND 3 AND 4

Key: TI = title, AB = abstract, KW = author supplied keywords

Appendix B

Characteristics of Included Studies

| Author (year) | *N* | #ES | Design | Name program | Abuse type | #C | #T | Outcome |
| --- | --- | --- | --- | --- | --- | --- | --- | --- |
| Baker et al. (2012) | 80 | 2 | QE | My Body, My Boundaries | Sexual abuse | 6 | 2 | K |
| Bustamente et al. (2019) | 496 | 1 | Cluster RCT | I have the right to feel safe at all times | Sexual abuse | 10 | 2 | K |
| Cecen-Erogul et al. (2013) | 36 | 1 | QE | A psycho-educational training program | Sexual abuse | 6 | 5 | K |
| Chen et al. (2012) | 46 | 4 | RCT | A CSA prevention program | Sexual abuse | 7 | 3 | K+S |
| Citac Tunc et al. (2018) | 83 | 7 | Cluster RCT | BST | Sexual abuse | 7 | 4 | K+S |
| Conte et al. (1985) | 20 | 4 | Cluster RCT | A CSA prevention program | Sexual abuse | 7 | 2 | K |
| Czerwinski et al. (2018) | 291 | 20 | QE | IGEL | Sexual abuse | 5 | 2 | K+S |
| Daigneault et al. (2012) | 160 | 2 | Cluster RCT | ESPACE workshop | Sexual abuse, verbal and physical violence | 6 | 3 | K+S |
| Dake et al. (2003) | 341 | 1 | QE | A child abuse prevention curriculum | Physical abuse, sexual abuse, emotional abuse, neglect, domestic violence. | 7 | 3 | K |
| Dhooper & Schneider (1995) | 796 | 1 | QE | Kids on the block: puppets and skits | Physical and sexual abuse | 1 | 3 | K |
| Hazzard et al. (1991) | 399 | 8 | QE | Feeling Yes, Feeling No | Sexual abuse | 8 | 5 | K+S |
| Hébert et al. (2001) | 133 | 3 | Cluster RCT | ESPACE program | Sexual abuse, verbal and physical violence | 4 | 3 | K+S |
| Jin et al. (2017) | 325 | 8 | RCT | -No name- | Sexual abuse | 5 | 2 | K+S |
| Kim et al. (2017) | 89 | 2 | QE | C-SAPE | Sexual abuse | 6 | 7 | K+S |
| Ko & Cosden (2001) | 137 | 4 | QE | CALM | Physical abuse, sexual abuse, emotional abuse, neglect. | 9 | 4 | K+S |
| Kolko et al. (1987) | 349 | 25 | QE | The Red Flag/Green Flag program | Sexual abuse | 5 | 7 | K+S |
| Kolko et al. (1989) | 248 | 10 | QE | The Red Flag/Green Flag program | Sexual abuse | 6 | 7 | K+S |
| Krahé & Knappert (2009) | 148 | 4 | Cluster RCT | (No) Child’s Play | Sexual abuse | 2 | 3 | S |
| MacIntyre & Carr (1999) | 727 | 1 | QE | Stay Safe Program | Sexual abuse | 3 | 2 | S |
| Moreno-Manso et al. (2014) | 317 | 40 | QE | -No name- | Physical abuse, sexual abuse, emotional abuse, neglect. | 11 | 6 | K |
| Morris et al. (2017) | 1176 | 5 | Cluster RCT | Safe@Last program | Sexual abuse | 7 | 4 | K |
| Nickerson et al. (2019) | 2172 | 3 | Cluster RCT | Second Step CPU | Sexual abuse | 5 | 5 | K+S |
| Ogunfowokan & Fajemilehin (2012) | 200 | 4 | QE | SAPEP | Sexual abuse | 1 | 2 | K |
| Oldfield et al. (1996) | 1269 | 3 | Cluster RCT | Project Trust | Sexual abuse | 2 | 3 | K+S |
| Pulido et al. (2015) | 67 | 1 | Cluster RCT | Safe Touches | Sexual abuse | 6 | 3 | K |
| Saslawsky & Wurtele (1986) | 67 | 2 | QE | Touch (film) | Sexual abuse | 5 | 2 | K+S |
| Taal & Edelaar (1997) | 292 | 15 | QE | Right to Security | Sexual abuse | 5 | 2 | S |
| Telljohann et al. (1997) | 431 | 2 | RCT | Sexual Abuse Prevention Program, 3^th^ Grade Curriculum | Sexual abuse | 4 | 4 | K+S |
| Tutty (1992) | 200 | 3 | RCT | Who Do You Tell program | Sexual abuse | 3 | 4 | K |
| Tutty (1997) | 231 | 3 | QE | Community Child Abuse Prevention Program | Sexual abuse | 2 | 1 | K |
| Weatherley et al. (2012) | 441 | 22 | QE | The Keeping Me Safe | Physical and sexual abuse | 8 | 3 | K+S |
| White et al. (2018) | 611 | 18 | Cluster RCT | Learn to be safe with Emmy and friends | Sexual abuse | 4 | 0 | K+S |
| Wolfe et al. (1986) | 290 | 1 | QE | -No name- | Physical and sexual abuse | 4 | 2 | K |
| Wurtele, Gillispie, et al. (1992) | 61 | 14 | RCT | BST | Sexual abuse | 6 | 4 | K+S |
| Wurtele, Kast, et al. (1992) | 85 | 6 | RCT | BST | Sexual abuse | 6 | 4 | K |
| Yom & Eun (2005) | 79 | 1 | RCT | CD-ROM educational program | Sexual abuse | 1 | 4 | K |
| Zhang et al. (2014) | 150 | 7 | QE | BST | Sexual abuse | 5 | 4 | K+S |

*Note.* *N* = total sample size; #ES = amount of effect sizes; Design = research design; Abuse type = type of abuse the program is aimed at; #C = amount of components addressed by school program; #T = amount of delivery techniques used in school program; Outcome = outcome measure; RCT = randomized controlled trial; QE = quasi-experimental; CSA = child sexual abuse; C-SAPE = Child Sexual Abuse Prevention Education; CALM = Child Abuse Listening and Mediation; CPU = Child Protection Unit; SAPEP = Sexual abuse prevention education package; BST = Body Safety Training; K= child abuse related knowledge; S = self-protection skills.
